# Comparing sparse inertial sensor setups for sagittal-plane walking and running reconstructions

**DOI:** 10.3389/fbioe.2025.1507162

**Published:** 2025-02-19

**Authors:** Eva Dorschky, Marlies Nitschke, Matthias Mayer, Ive Weygers, Heiko Gassner, Thomas Seel, Bjoern M. Eskofier, Anne D. Koelewijn

**Affiliations:** ^1^ Machine Learning and Data Analytics Lab, Department Artificial Intelligence in Biomedical Engineering (AIBE), Friedrich-Alexander-Universität Erlangen-Nürnberg (FAU), Erlangen, Germany; ^2^ Institute of Mechatronic Systems, Leibniz Universität Hannover, Hannover, Germany; ^3^ Department of Molecular Neurology, University Hospital Erlangen, Erlangen, Germany; ^4^ Digital Health and Analytics, Fraunhofer Institute for Integrated Circuits IIS, Erlangen, Germany; ^5^ Translational Digital Health Group, Institute of AI for Health, Helmholtz Zentrum München - German Research Center for Environmental Health, Neuherberg, Germany; ^6^ Chair of Autonomous Systems and Mechatronics, Department of Electrical Engineering, Friedrich-Alexander-Universität Erlangen-Nürnberg (FAU), Erlangen, Germany

**Keywords:** gait analysis, gait simulations, inertial measurement units, optimal control, trajectory optimization

## Abstract

Estimating spatiotemporal, kinematic, and kinetic movement variables with little obtrusion to the user is critical for clinical and sports applications. One possible approach is using a sparse inertial sensor setup, where sensors are not placed on all relevant body segments. Here, we investigated if movement variables can be estimated similarly accurate from sparse sensor setups as from a full lower-body sensor setup. We estimated the variables by solving optimal control problems with sagittal plane lower-body musculoskeletal models, in which we minimized an objective that combined tracking of accelerometer and gyroscope data with minimizing muscular effort. We created simulations for 10 participants at three walking and three running speeds, using seven sensor setups with between two and seven sensors located at the feet, shank, thighs, and/or pelvis. We found that differences between variables estimated from inertial sensors and those from optical motion capture were small for all sensor setups. Including all sensors did not necessarily lead to the smallest root mean square deviations (RMSDs) and highest coefficients of determination ( ${\text{R}}^{2}$ ). Setups without a pelvis sensor led to too much forward trunk lean and inaccurate spatiotemporal variables. Mean RMSDs were highest for the setup with two foot-worn inertial sensors (largest error in knee angle during running: 18 deg vs. 11 deg for the full lower-body setup), and ranged between 4.8–18 deg for the joint angles, between 1.0–5.4 BW BH% for the joint moments, and between 0.03 BW–0.49 BW for the ground reaction forces. We found strong or moderate relationships ( ${\text{R}}^{2}>0.5$ ) on average for all kinematic and kinetic variables, except for the hip and knee moment for five out of the seven setups. The large range of the coefficient of determination for most kinetic variables indicated individual differences in simulation quality. Therefore, we conclude that we can perform a comprehensive sagittal-plane motion analysis with sparse sensor setups as accurately as with a full sensor setup with sensors on the feet and on either the pelvis or the thighs. Such a sparse sensor setup enables comprehensive movement analysis outside the laboratory, by increasing usability of inertial sensors.

## 1 Introduction

Gait analysis is a fundamental tool for understanding human locomotion, but is currently limited to laboratory environments. Gait analysis can include spatiotemporal, kinematic, and kinetic variables. Spatiotemporal variables provide information about the movement quality and can be used for clinical diagnoses (e.g., Jakob et al., 2021). Kinematic, i.e., joint angles, and kinetic information, i.e., ground reaction forces (GRFs), joint moments, muscle forces, as well as variables calculated from those, such as joint reaction forces, are required to understand the mechanical and physiological mechanisms of human movement. Therefore, a comprehensive analysis, including spatiotemporal, kinematic, and kinetic variables, is important in sports performance assessments (e.g., Dorschky et al., 2019a; Mohr et al., 2024) and for rehabilitation (e.g., Kim, 2017). A measurement method that is cost-effective and allows for recordings “in the wild,” or outside the laboratory environment, could enable widespread use of gait analysis in different clinical and sports applications. Such methods could for example be markerless motion capture, based on video images, or motion capture using inertial measurement units (IMUs), and possibly other wearable sensors. Video-based motion capture (Uhlrich et al., 2023; Nakano et al., 2020) still requires the person to be in the camera’s field of view. Moreover, lighting conditions, camera placement, and occlusion can affect the accuracy, limiting the flexibility of the system. IMUs are small, low cost, wearable sensors that contain an accelerometer and a gyroscope, and sometimes a magnetometer (Zhang et al., 2013), which can also be combined with other wearable sensors, such as pressure insoles (Carter et al., 2024). However, combining different sensor systems requires time synchronization, which is challenging (Kugler et al., 2012). IMUs are attached to different body segments to measure their linear accelerations and angular velocities, as well as possible other signals. Thus, IMU-based motion capture can be used to measure movement outside the lab in any environment (Fleron et al., 2019).

While many different methods have been developed to estimate spatiotemporal variables (see Kobsar et al., 2020 for a review) and kinematics (see Weygers et al., 2020 for a review) of movements from inertial sensor data, estimation of kinetics is more challenging. Joint moments are commonly computed using inverse dynamics when both kinematics and ground reaction forces (GRFs) are estimated from the IMU data (Ancillao et al., 2018; Karatsidis et al., 2019; Muller et al., 2020; Diraneyya et al., 2021). However, this step-wise approach leads to error propagation. Errors in kinematic estimates can arise from factors such as soft tissue artifacts and the drift that occurs due to the numerical integration of IMU signals over time. Errors can also exist in GRF estimates, e.g. due to load sharing assumptions between the feet. These errors directly affect the results of the inverse dynamics calculations. Therefore, we aim to simultaneously estimate spatiotemporal, kinematic and kinetic variables from raw IMU data, which can be done using machine learning (e.g., Mundt et al., 2020; Yi et al., 2021; Conte Alcaraz et al., 2021; Mundt et al., 2021; Dorschky et al., 2020) or using optimal control (e.g., Dorschky et al., 2019a; Nitschke et al., 2024). Machine learning models can be trained to directly map IMU data to biomechanical variables (e.g., Dorschky et al., 2020; Mundt et al., 2020; Yi et al., 2021; Conte Alcaraz et al., 2021; Mundt et al., 2021; Bicer et al., 2022). However, an important limitation of machine learning models is that it cannot be proven that the resulting machine learning models outputs dynamically consistent results, meaning that the kinematic and kinetic variables might not follow the laws of physics. Furthermore, machine learning models are typically trained and tested using lab-based optical motion capture (OMC) and IMU data, which means that there is no proof that such a model will perform well “in the wild.” Another major challenge when applying machine learning to estimate biomechanical variables is the availability of training data, since the machine learning model accuracy has been shown to improve with the size of the training dataset (Rapp et al., 2021).

In order to obtain interpretable and dynamically consistent motions, we have developed an optimal control approach to simultaneously estimate the kinetics and kinematics of walking and running based on raw accelerometer and gyroscope measurements (Dorschky et al., 2019b). With this approach, we find a dynamics simulation for a musculoskeletal model, such that virtual inertial sensor data of the simulation match the recorded inertial sensor data as closely as possible. This approach does not require a training dataset, inherently overcomes the aforementioned drift problems, and can mitigate soft-tissue artifacts (Dorschky et al., 2019b). An optimal control problem is solved to find a simulation for a sagittal plane musculoskeletal model that minimizes effort while also minimizing a tracking error between virtual and measured inertial sensor data, specifically angular velocities and linear accelerations. The resulting simulation contains spatiotemporal, kinetic, and kinematic variables that are dynamically consistent (Nitschke et al., 2023), meaning that the simulation follows the laws of physics as they are described in the optimal control problem. The musculoskeletal model used in the optimal control problem represents the human who was measured. Therefore, we know that the resulting motion is close to that of this person, although there are differences due to modeling assumptions. Since we use a musculoskeletal model, the kinetic variables are the GRFs, joint moments and muscle forces. We include muscle dynamics to constrain torques to be physiologically realistic. Furthermore, the tracking approach avoids integration of the inertial sensor measurements and thus the related integration drift. We previously showed that with this approach, we could estimate joint angles and joint moments similar (joint angles: mean Pearson’s correlation at least 0.96, mean root mean square deviation (RMSD) between 4.3–8.7 deg, joint moments: mean Pearson’s correlation at least 0.76, mean RMSD between 1.5–3.4 BW BH%, GRF: mean Pearson’s correlation at least 0.94, RMSD between 4.1–32.0 BW %) to those obtained through inverse kinematics and inverse dynamics based on OMC and GRF measurements (Dorschky et al., 2019b).

However, estimating both kinetics and kinematics from inertial sensor data commonly requires a sensor on each body segment of interest. For example, to estimate leg kinematics and kinetics using optimal control, we used 7 inertial sensors (Dorschky et al., 2019b). Others used 8 sensors to include the trunk (Al Borno et al., 2022), or 17 sensors for a full-body analysis (Karatsidis et al., 2019). When more sensors are placed on the body, the wearer, and thus their motion, can be increasingly affected (Schlachetzki et al., 2017). Furthermore, using more sensors increases cost, reduces practicality, and increases the chance of errors in IMU placement (Gholami et al., 2020). Therefore, our aim is to create an IMU-based motion analysis approach that is as unobtrusive as possible by reducing the number of necessary sensors. Sparse sensor sets have been developed to estimate individual gait variables, such as spatiotemporal (e.g., Jakob et al., 2021), kinematic (Li et al., 2020; Gholami et al., 2020; Hossain et al., 2022; Von Marcard et al., 2017), three dimensional kinematic (Von Marcard et al., 2017), or kinetic (Li et al., 2021) variables. Furthermore, neural networks have been investigated to estimate specific quantities of interest using only two IMUs on the shanks (Gholami et al., 2020) or the feet (Jakob et al., 2021; Hossain et al., 2022). Others have proposed physics-based optimization to track the sensor orientation with a torque-driven model (Li et al., 2021) or a human body shape model (Von Marcard et al., 2017). However, these approaches do not provide a comprehensive analysis including various biomechanical variables, or neglect physical correctness or muscle dynamics. Furthermore, to our knowledge, no systematic comparison has been performed to evaluate different sensor configurations for estimating spatiotemporal variables, kinematics, and kinetics during walking and running.

In this work, we performed such a systematic comparison to evaluate how well spatiotemporal variables, joint moments, GRFs, and joint angles can be estimated from sparse sensor setups using an optimal control approach. Previously, we developed an optimal control approach for a full lower-body sensor setup (Dorschky et al., 2019b). Since realistic walking simulations of musculoskeletal models can also be created by solving optimal control problems with no data tracking (Falisse et al., 2019), we expect that we can also apply the approach developed by Dorschky et al. (2019b) to sparse sensor setups as well. Here, we therefore investigated the accuracy of reconstructive simulations created by solving optimal control problems for six sparse sensor setups and the full lower-body setup. We created the six sparse sensor setups by varying the number of IMUs included, as well as the body segments on which they were attached. All sensor setups included sensors at the most distal segment (the feet) to ensure that measurements are available for all leg segments. We used a minimal setup of two IMUs placed on the feet, three setups with sensors on two locations: feet and shanks, feet and thighs, and feet and pelvis, and two setups with sensors at three locations: feet, shanks and pelvis and feet, thighs and pelvis, and compared those to a full lower-body setup with sensors on the feet, shanks, thighs and pelvis. We then created optimal control simulations of walking and running for each sensor setup and investigated the difference between the resulting spatiotemporal variables, gait kinematics, and gait kinetics for each sparse sensor setup with those obtained using OMC and using the full lower-body lower-body sensor setup as used by Dorschky et al. (2019b).

## 2 Methods

**FIGURE 1:**
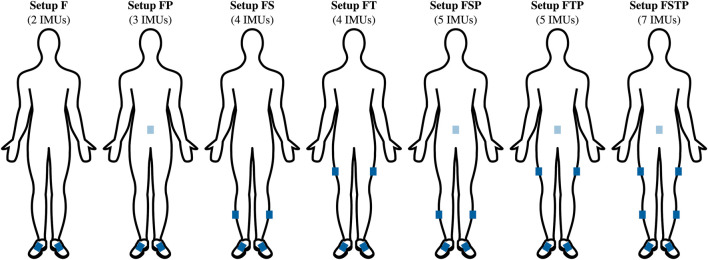
Seven sensor setups. The blue rectangles indicate the approximate location of the inertial measurement units (IMUs). The sensor setups are abbreviated using the first letter of the segments equipped with a sensor: F-feet, S-shanks, T-thighs, P-pelvis. The setup FSTP corresponds to a full lower-body sensor setup with seven IMUs.

We reconstructed walking and running motions of a sagittal-plane musculoskeletal model using raw inertial sensor measurements, i.e., raw gyroscope and accelerometer data, from six different sparse sensor setups and one full lower-body sensor setup (see Figure 1). Sensors were placed symmetrically for all setups, and each setup included sensors at the feet. We included feet sensors in all setups, since these sensors can be attached to the shoe, and are therefore unobtrusive, while they have also provided reliable information in past studies (Jakob et al., 2021). For each sensor setup, we created musculoskeletal model simulations by solving optimal control problems that minimized the difference between measured and virtual sensor data. To evaluate the different sensor setups and their respective simulations, we compared the difference between these simulations and an OMC analysis.

### 2.1 Experimental data

We used measured data previously recorded with seven custom-built IMUs (Portabiles HealthCare Technologies GmbH, Erlangen, Germany) placed on the pelvis, legs and feet (Figure 1, setup FSTP) and an OMC system including one force plate. Dorschky et al. (2019b) described the data recording and data pre-processing and used this dataset to evaluate optimal control simulations from the full lower-body sensor setup (setup FSTP). In this work, we evaluated six sensor setups with sparser sensor placement (Figure 1, setup F, FP, FS, FT, FSP, and FTP), and compared those to the full lower-body sensor setup as used by Dorschky et al. (2019b) and OMC data. The raw and pre-processed data (mean and standard deviation over 10 trials) are available in Dorschky et al. (2024).

This dataset contained recordings of 10 healthy male participants (age: 27.1 ± 2.6 years, height: 181.9 ± 5.3 cm, weight: 76.9 ± 8.6 kg) at six different speeds (slow walking: 0.9–1.0 m s^−1^; normal walking: 1.3–1.4 m s^−1^; fast walking: 1.7–1.8 m s^−1^; slow running: 3.1–3.3 m s^−1^; normal running: 3.9–4.1 m s^−1^; fast running: 4.7–4.9 m s^−1^). Participant recruitment and data collection took place from June to August 2016. All participants gave their informed written consent prior to participation. The study was conducted in line with the ethical principles of the Declaration of Helsinki and it was approved by the ethics committee of the Medical Faculty at the FAU Erlangen-Nürnberg, Germany (Ref.-No.: 106_13 B).

As described by Dorschky et al. (2019b), IMU axes were aligned with the body segment axes using functional calibration movements. From the OMC-data, sagittal plane joint angles and joint moments were calculated using the GaitAnalysisToolkit [see Moore et al., 2014) according to Winter (2009)]. IMU and OMC measurements were synchronized using a custom flash trigger system. In addition, we corrected for a small time offset (approx. 20 ms) between IMU and OMC measurements, which was determined by calculating the cross-correlation between the time derivative of the hip, knee, and ankle joint angles obtained from the OMC analysis and the angular velocity measured by the adjacent gyroscopes.

### 2.2 Musculoskeletal model and dynamics

**FIGURE 2:**
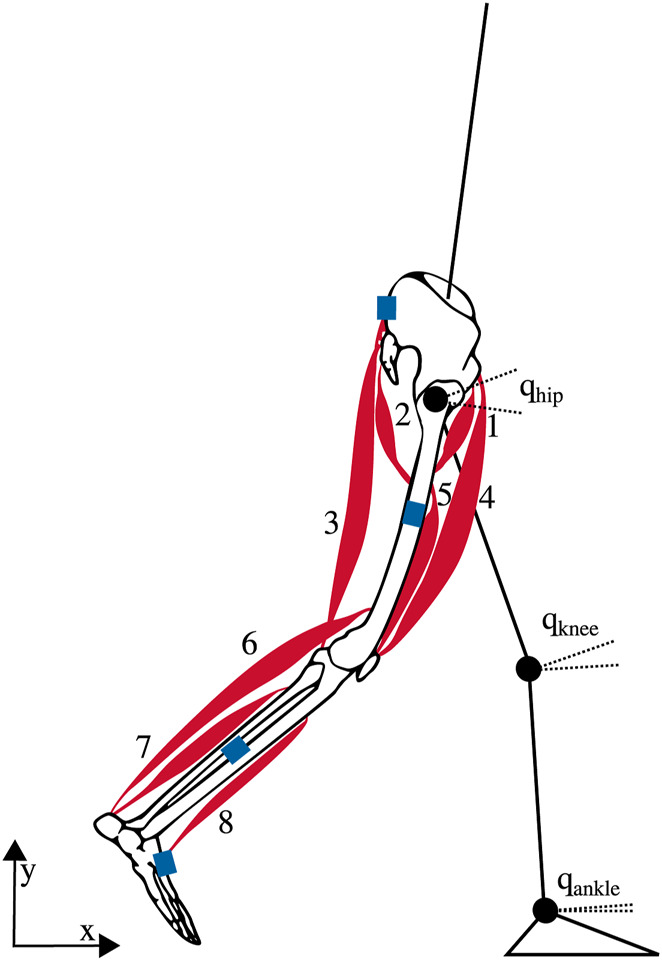
Musculoskeletal model with seven rigid segments and 16 Hill-type-muscles, eight per leg: 1 – iliopsoas, 2 – glutei, 3 – hamstrings, 4 – rectus femoris, 5 – vasti, 6 –gastrocnemius, 7 – soleus, and 8 – tibialis anterior (Dorschky et al., 2019b; Van den Bogert et al., 2012). The blue rectangles indicate the approximate location of the inertial measurement units (IMUs).

We used a sagittal-plane musculoskeletal model to create our gait simulations (Dorschky et al., 2019b; Van den Bogert et al., 2012). The skeleton consisted of seven segments: head-arms-trunk (HAT), two upper legs, two lower legs, and two feet. It was described by nine generalized coordinates q : the position and orientation of the trunk, two hip angles, two knee angles, and two ankle angles (Figure 2). We personalized the musculoskeletal model’s segment masses, lengths, center of mass locations and moments of inertia based on the study participants’ full-body height and weight (Winter, 2009). We modeled 16 lower-leg muscles as three-element Hill-type muscles (Van den Bogert et al., 2011) (Figure 2). Overall the model’s state, x(t) , was described by the nine generalized coordinates q(t) , the corresponding nine velocities v(t) , 16 contractile element lengths $l_{CE}\left(t\right)$ , and 16 muscle activations a(t) : $x\left(t\right)≔{\left[q\left(t\right)\;v\left(t\right)\;l_{CE}\left(t\right)\;a\left(t\right)\right]}^{T}$ for 0≤t≤T with movement duration T .

We modelled contact between the musculoskeletal model and the ground using two contact points at each foot (heel and toe) (Dorschky et al., 2019b). We used a penetration-based contact model to describe the vertical GRF, and a friction model for the horizontal GRF. The contact model equations are further described in (Todorov, 2010). Each contact point was described by its global (x,y) position, and the anterior-posterior $\left(F_{x}\right)$ and vertical $\left(F_{y}\right)$ force.

We defined the dynamics by combining the musculoskeletal model and the contact model. The model was controlled through the 16 muscle excitations, u(t) for 0≤t≤T (Dorschky et al., 2019b). The system dynamics were fully described by implicit differential equations as a function of x(t) , $\dot{x}\left(t\right)$ and u(t) : $f\left(x\left(t\right),\dot{x}\left(t\right),u\left(t\right)\right)=0$ . The system dynamics, including multibody dynamics, muscle-tendon equilibrium, activation dynamics, and contact model, f() , were twice differentiable with respect to all x , $\dot{x}$ and u (Van den Bogert et al., 2011), such that the optimal control problems can be solved using a gradient-based optimization algorithm.

We calculated virtual IMU data using a virtual sensor model that was added to the musculoskeletal model (Dorschky et al., 2019b). We placed virtual sensors in their respective position, as measured during the experiment, on the musculoskeletal model, and calculated the virtual sagittal plane accelerometer and gyroscope data from the model state and its derivative (Dorschky et al., 2019b). The gyroscope signal represented the angular velocity of the respective body segment relative to the global coordinate system and was derived from the generalized velocities. The acceleration signal represented the linear acceleration at the respective sensor position on the body segment relative to the global coordinate system and was a combination of the acceleration of the body segment origin and the acceleration caused by the segment rotation. The used equations can be found in (Dorschky et al., 2019b).

### 2.3 Optimal control problems

We created gait simulations from inertial sensor data by solving optimal control problems. In these optimal control problems, a multi-objective optimization was solved to find a periodic walking or running cycle that minimized muscular effort, while also tracking the measured inertial sensor data. We constructed the tracking objective to minimize the squared difference between the measured accelerations and angular velocities from the IMUs and the corresponding simulated accelerations and angular velocities from the virtual sensor model, averaged over the gait cycle duration T

$$\begin{array}{ll} J_{\text{track}}\left(x\left(t\right),u\left(t\right)\right) & =\frac{1}{T\left(3n_{\text{IMU}}\right)}\int_{t=0}^{T}\overset{n_{\text{IMU}}}{\underset{j=1}{\sum }}\left({\left(\frac{a_{j,x}\left(x\left(t\right)\right)-\mu_{a_{j,x}}\left(t\right)}{\sigma_{a_{j,x}}\left(t\right)}\right)}^{2}\right.\\ & \;+\left.{\left(\frac{a_{j,y}\left(x\left(t\right)\right)-\mu_{a_{j,y}}\left(t\right)}{\sigma_{a_{j,y}}\left(t\right)}\right)}^{2}+{\left(\frac{\omega_{j,z}\left(x\left(t\right)\right)-\mu_{\omega_{j,z}}\left(t\right)}{\sigma_{\omega_{j,z}}\left(t\right)}\right)}^{2}\right)dt, \end{array}$$

where the number of IMUs $n_{\text{IMU}}$ ranged between two and seven depending on the setup in Figure 1. Since we used a two-dimensional musculoskeletal model defined in the x - y -plane, we calculated the virtual accelerations in x - and y -direction $a_{j,x}$ and $a_{j,y}$ , and the angular velocity $\omega_{j,z}$ around the z -axis for each IMU $j\in \left\{1,\ldots ,n_{\text{IMU}}\right\}$ (Dorschky et al., 2019b). We tracked experimental IMU data μ averaged over ten gait cycles. The squared difference between simulated and measured signals was divided by the variance of the measured data $\sigma^{2}$ . This approach leads to a smaller weight being applied when there is a large variance, e.g., due to soft tissue artefacts, and automatically provides an appropriate weighting between different sensor modalities. In our case, the duration of a gait cycle T was equal to the average duration of the ten gait cycles.

We computed muscular effort as squared muscle excitations averaged over the duration T and normalized to the squared walking or running speed v

$$J_{\text{effort}}\left(x\left(t\right),u\left(t\right)\right)=\frac{1}{Tv^{2}n_{\text{mus}}}\int_{t=0}^{T}\overset{n_{\text{mus}}}{\underset{i=1}{\sum }}u_{i}^{2}\left(t\right)dt.$$

These objective terms yielded the following optimal control problem (Van den Bogert et al., 2011; Dorschky et al., 2019b)

$$\underset{x\left(0\right),u\left(t\right),v}{\text{minimize}}\;J\left(x\left(t\right),u\left(t\right)\right)=J_{\text{track}}\left(\cdot \right)+W_{\text{effort}}J_{\text{effort}}\left(\cdot \right)+W_{\text{reg}}J_{\text{reg}}\left(\cdot \right)$$

$$\text{subject to}\;\;f\left(x\left(t\right),\dot{x}\left(t\right),u\left(t\right)\right)=0\;\text{for }0\leq t\leq T\;\left(\text{system dynamics}\right)$$

$$\;\;x_{L}\leq x\left(t\right)\leq x_{U}\;\;\text{for }0\leq t\leq T\;\;\left(\text{bounds on states}\right)$$

$$\;\;u_{L}\leq u\left(t\right)\leq u_{U}\;\;\text{for }0\leq t\leq T\;\;\left(\text{bounds on controls}\right)$$

$$\;\;\;x\left(T\right)=x\left(0\right)+vTx_{\mathrm{hor}}\;\;\left(\text{periodicity}\right),$$ (1)

where $W_{\mathrm{effort}}=300$ and $W_{\text{reg}}=10^{-5}$ are the weightings of the effort term and regularization term, respectively. These weights were the same as those used in Dorschky et al. (2019a). In Equation 1, we enforced a periodic forward motion, where $x_{\mathrm{hor}}$ is a vector of states to which a horizontal displacement applies, which are the horizontal pelvis position and the horizontal contact point positions.

To solve the optimal control problems, we transcribed them (objectives and constraints) using direct collocation with a backward Euler discretization and N=100 time nodes (Dorschky et al., 2019b; Van den Bogert et al., 2011). We added an extra collocation node N+1 to evaluate our periodicity constraint (Equation 1), such that the state at node N+1 should be the same as at node 1 with a horizontal translation. Our decision variables consist of the model’s state, x , and input, u at each time node, as well as the global contact point locations and forces. We chose to include these, since preliminary work showed that this approach speeds up the optimization. We added constraints to ensure that the location and forces match those calculated from the contact model using the model’s state. Furthermore, our decision variables also included the motions’s speed and duration. In total, 8,284 variables (101 time nodes x (18 multibody states + 32 muscle states + 16 muscle stimulations + 4 x 4 contact point decision variables) + speed + duration = 8,284) were optimized with 6,682 constraints (100 time nodes x (18 multibody dynamics equations + 32 muscle dynamics equations + 4 x 4 contact model equations) + 82 periodicity constraints = 6,682). We used the same initial guess and the same objective weights as (Dorschky et al., 2019b). We solved the resulting large scale nonlinear optimization problems with IPOPT with MUMPS (Wächter and Biegler, 2006). We solved 420 optimal control problems, i.e., simulations for seven setups for 10 participants at six speeds, on a computer cluster using one Intel Xeon E3-1240 v6 for each simulation.

### 2.4 Data analysis

We analyzed the convergence and computation time of the optimal control problems, followed by a comparison of the spatiotemporal variables (walking speed, stance time, stride length), the kinematic variables (sagittal plane joint angles), and the kinetic variables (sagittal plane joint moments and GRFs) as calculated with the OMC measurements and the IMU measurements. First, we determined the speed as the translation of the right heel divided by the duration of the gait cycle, the stance time as the ratio of the duration of the stance phase (Maiwald et al., 2009) and the duration of the gait cycle, and the stride length as the translation of the right heel.

We quantified the similarity of the spatiotemporal variables between the different IMU setups and the OMC setup using the Root mean square deviation (RMSD). For each walking and running speed, we calculated the RMSD between the results calculated with the different IMU setups and the results of the OMC analysis using the data of all participants. In addition, we generated scatter plots comparing the spatiotemporal variables obtained from the OMC measurements with those obtained from the different IMU setups in order to observe systematic differences between the setups.

Next, we compared kinetic and kinematic trajectories of the right leg between the results calculated with the different inertial sensor setups and the results of the OMC analyis using the coefficient of determination ( ${\text{R}}^{2}$ ) and RMSD. We calculated the coefficient of determination between the IMU data and the linear transform of the OMC data, which we determined using the linear fit method (LFM) (Iosa et al., 2014), and the RMSD for each walking or running cycle of each participant individually. Then, we determined the mean (using Fisher’s Z-transform for the square root of the coefficient of determination) over all walking and running cycles and participants. We computed the coefficient of determination and RMSD for the entire gait cycle for the joint angles and for the stance phase for the joint moments and GRFs.

## 3 Results

We solved 420 optimizations, which required a mean ± standard deviation CPU time of 48 ± 3 min (Table 1, Supplementary Material). 418 of the 420 simulations converged, while no optimal solution was found for two simulations. For one participant, the restoration phase failed for setup FP in the slow walking trial, while for another participant, the same happened for setup F in the fast walking trial. We removed these two simulations from the analysis. CPU times for walking simulations were higher than for running simulations, but similar for the different setups except for setup F for walking. We added the measured IMU trajectories and those calculated from the optimal control simulations in the Supplementary Material: Supplementary Figure S1 shows the averaged results for walking, Supplementary Figure S2 for running, and the other files show the individual results for each trial, participant, and sensor setup.

**TABLE 1:** CPU time in minutes for converged optimizations of the different inertial sensor setups (mean ± standard deviation).

Setup	F	FP	FS	FT	FSP	FTP	FSTP
Walking	110 ± 85	77 ± 33	61 ± 28	54 ± 20	63 ± 28	63 ± 22	68 ± 30
Running	27 ± 10	26 ± 9	27 ± 9	31 ± 11	28 ± 9	29 ± 10	34 ± 10

**FIGURE 3:**
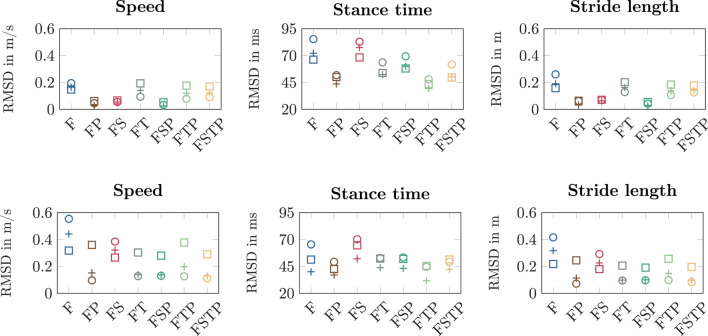
Root mean square deviation (RMSD) for speed, stance time, and stride length between the different inertial measurement unit (IMU) setups and the optical motion capture (OMC) for walking (top row) and running (bottom row). We calculated the RMSDs over all participants for each walking and running speed and each setup.

We found that the RMSDs of the spatiotemporal variables were similar between all sensor setups, though setup F performed worst (Figure 3). The RMSDs for speed and stride length were highest for setup F (speed^1^.: [0.15; 0.55] m/s ; stride length: [0.16; 0.42] m ) for most walking and running conditions. When adding a pelvis sensor (setup FP), RMSDs of speed and stride length decreased (speed: [0.03; 0.36] m/s ; stride length: [0.04; 0.24] m ). The RMSDs for speed and stride length were lower for the setups with a thigh sensor (setups FP, FS, and FSP), since speed and stride length were systematically underestimated when a thigh sensor was added (Figure 3). For the setups with sensors only on the lower leg segments (setups F and FS), the stance time RMSD was worst, up to 27.2 ms higher than the full lower-body sensor setup (setup FSTP).

**FIGURE 4:**
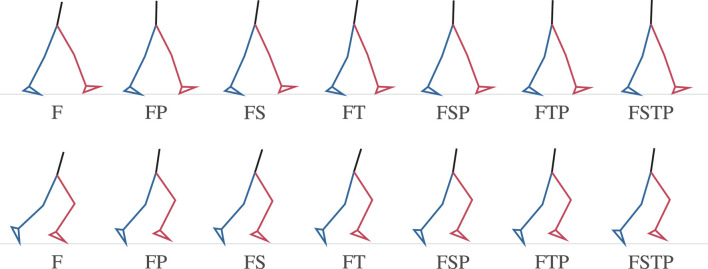
Stick figure representing the mean kinematics at the first time point of the different inertial measurement unit (IMU) setups for walking (top) and running (bottom) over all participants. The stick figures show that without a pelvis sensor (setups F, FS, and FT), forward trunk lean is too large.

When visualizing the mean of our simulations for each setup with stick figures, we found that the simulations without a pelvis sensor had more forward trunk lean than the full lower-body sensor setup (Figure 4). During walking, the mean forward trunk lean over all participants and over time was at least 7 deg for setups F, FS, and FT, while it was below 2 deg for all setups with a pelvis sensor. Similarly, for running, the mean forward trunk lean for setups F, FS, and FT was at least 16 deg, while it remained below 9 deg for the setups with a pelvis sensor. We also observed that the step length for setup F is larger than the step length of all other setups for walking and running, supporting the larger RMSDs shown in Figure 3 for the stride length.

**FIGURE 5:**
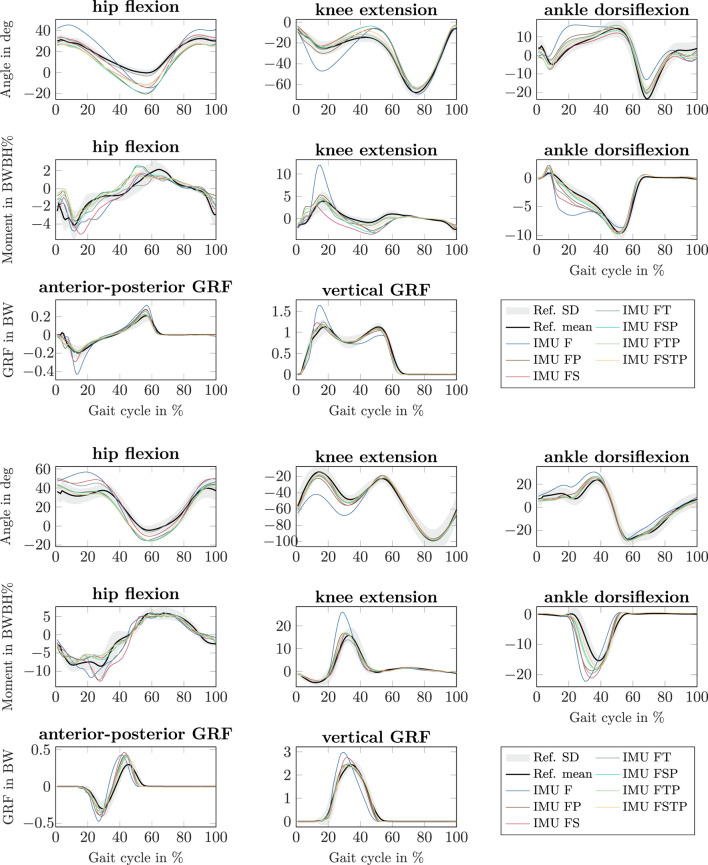
Sagittal-plane joint angles, joint moments, and ground reaction forces (GRFs) of the right lower limb for walking (top three rows, right heel strike to right heel strike) and running (bottom 3 row, left toe off to left toe off) at all speeds, from the different inertial measurement unit (IMU) setups (colored lines) and the references values from optical motion capture system and force plate data (mean: black line, standard deviation: grey fill). All lines represent the mean over all participants. Joint moments were scaled to bodyweight bodyheight percent (BW BH%) and GRFs were scaled to bodyweight (BW).

Our simulations showed that joint angle, joint moment, and GRF estimates generally benefitted from having sensors placed at the pelvis or thighs in addition to those at the feet, meaning that setups FP, FT, FSP, FTP and FSTP generally led to more accurate estimates than setup F (especially) and setup FS (Figure 5, Supplementary Material for individual results). For setup F, the mean peak knee flexion angle during stance was much higher than for all other setups for walking (47 deg for setup F vs. 22–30 deg for all other setups and 25 deg for the OMC result) and, to a lesser extent, for running (68 deg for setup F vs. 58–62 deg for all other setups and 49 deg for the OMC result), and so was the mean peak knee extension moment (walking: 12 BW BH% for setup F vs. 2.8–5.3 BW BH% for all other setups and 3.9 BW BH% for the OMC result, running: 26 BW BH% for setup F vs. 14–17 BW BH% for all other setups and 16 BW BH% for the OMC result). The mean peak ankle dorsiflexion angle was higher as well during running (30 deg for setup F) compared to the other setups (24–27 deg) and the OMC result (24 deg). For setups without thigh sensors (F, FP, FS, and FSP), we also observed that the mean hip range of motion was larger than in the OMC result for the walking simulations. For setups F, FP, FS, and FSP, the mean hip range of motion was at least 47 deg, while it was between 37–40 deg for the other setups and 32 deg for the OMC result. Similarly, we observed a larger mean peak flexion moment in the knee, which was equal to at least 2.7 BW BH% for setups F, FP, FS, and FSP, while it was between 1.2–2.0 BW BH% for the other setups and 1.3 BW BH% for the OMC result. The mean forward progression of the ankle dorsiflexion was faster than the OMC result for all setups except setup FS for the walking simulations (e.g., on average 5 deg dorsiflexion was reached at 21% of the gait cycle in the OMC result and at 23% for setup FS, but between 12% for setup F and 19% for all other setups), while a similar fast progression was observed in the ankle plantarflexion moment for setups F, FP, FT, and FS, both for walking and running. The mean peak hip flexion moment during walking occurred earlier in the gait cycle than in the OMC result for all setups except setup FT (62% of the gait cycle for setup FT and the OMC result vs. 50%–54% for all other setups), while for running, it occurred earlier for setups FT and FTP (59% of the gait cycle for setups FT and FTP vs. 65% for the OMC result and 67% for all other setups). During running, the mean peak hip extension moment was larger for the setups without a pelvis sensor (12–13 BW BH% for setups F, FS, and FT) than the other setups (7–8 BW BH%) and the OMC result (9 BW BH%). Furthermore, the mean anterior-posterior GRF displayed larger braking and push-off forces for all IMU setups, most prominently for setup F (e.g., mean peak braking force during walking: 0.2 BW for the OMC result vs. 0.32 BW for setup F and 0.22–0.28 BW for all other setups), while the mean peak vertical GRF was larger during walking for setup F (1.6 BW) than all other setups and the OMC result (1.1–1.2 BW) and larger during running for setups F and FS (F: 3.0 BW and FS: 2.8 BW) than for all other setups and the OMC result (2.3–2.5 BW).

**FIGURE 6:**
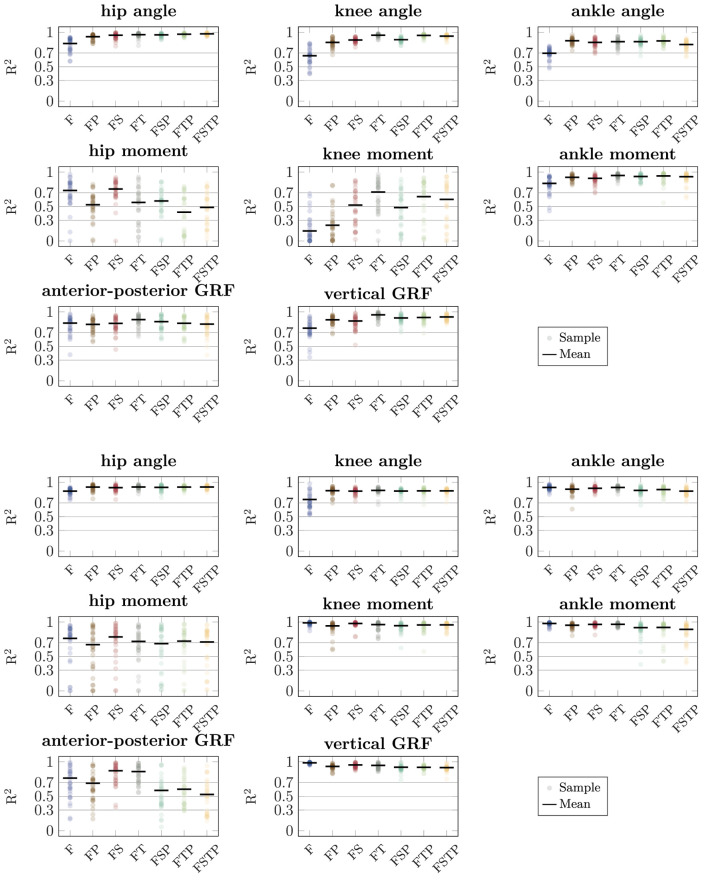
Coefficient of determination ( ${\text{R}}^{2}$ ) for the sagittal-plane joint angles, joint moments, and ground reaction forces (GRFs) between the different inertial measurement unit (IMU) setups and references values from optical motion capture system and force plate data for walking (top three rows) and running (bottom three rows), determined using the linear fit method. The circles show the coefficient of determination for each walking and running cycle of each participant. The bar shows the mean value over all cycles and participants computed using Fisher’s Z-transform on the square root of the coefficient. The horizontal lines categorize the relationship as strong ( ${\text{R}}^{2}>0.7$ ), moderate ( 0.5< ${\text{R}}^{2}<0.7$ ), weak ( 0.3< ${\text{R}}^{2}<0.5$ ), and no ( ${\text{R}}^{2}<0.3$ ).

Using the LFM, we found strong relationships ( ${\text{R}}^{2}>0.7$ ) on average for the joint angles (except for setup F), the ankle moment, and the vertical GRF, and weak ( 0.3< ${\text{R}}^{2}<0.5$ ) to no ( ${\text{R}}^{2}<0.3$ ) relationships for the hip moment and knee moment during walking, while the variation between participants was high in the kinetic variables (Figure 6). For running, most relationships were strong on average, but we found moderate relationships ( 0.5< ${\text{R}}^{2}<0.7$ ) on average for the hip moment (setup FP and FSP) and horizontal GRF (setup FP, FSP, FTP, and FSTP). For walking, most relationships for the joint angles were strong on average, except for the joint angles for setup F, where the relationship was moderate on average. The relationship for the hip moment for walking was strong on average only for setup F and setup FS, while it was weak for setups FTP and FSTP, and moderate otherwise. The relationship for the knee moment for walking was strong on average only for setup FT, moderate on average for setup FS, FTP, and FSTP, weak on average for setup FSP, and no relationship was found for setup F and FP. The largest range of coefficients of determination for the joint angles was 0.45 (setup F, knee angle, running, 0.53–0.98), while the highest range for any other setup was 0.35 for setup FP (ankle angle, running), and the highest range for setup FSTP 0.27 (ankle angle, walking). The range for the hip moment was large for all setups (at least 0.79 for setup FTP, walking), as well as for the knee moment during walking (at least 0.69, setup F), while for the ankle moment, it was between 0.08 (setup FT, running) and 0.61 (setup FSP, running). The vertical GRF had the smallest range for setup F during running (0.04) and the largest range for setup F during walking (0.60), while the horizontal GRF had a larger range for running (at least 0.43 for setup FT) than for walking (at most 0.59 for setup F).

**FIGURE 7:**
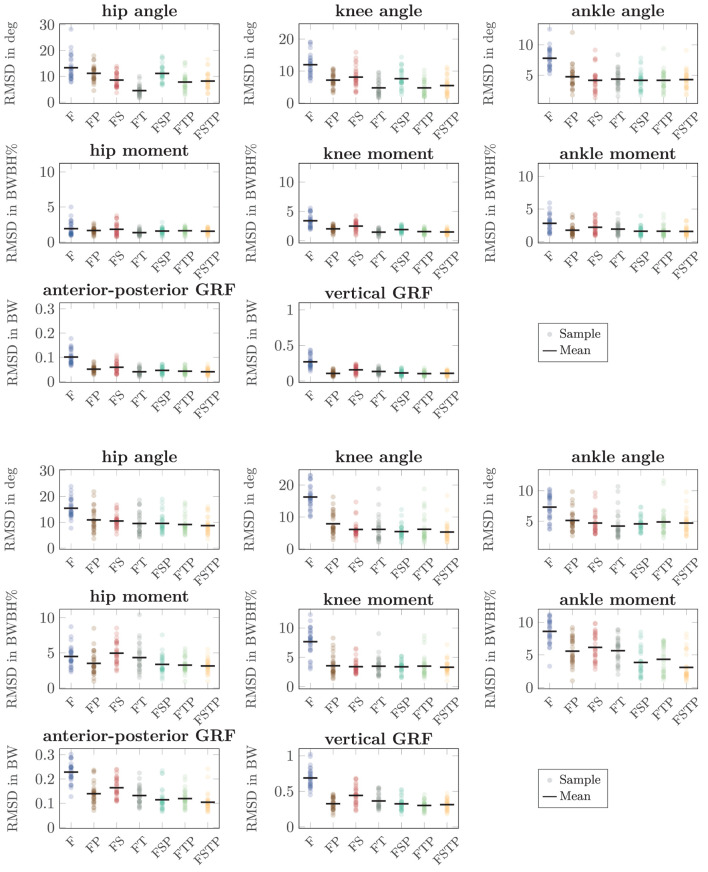
Root mean square deviation (RMSD) for the sagittal-plane joint angles, joint moments, and ground reaction forces (GRFs) between the different inertial measurement unit (IMU) setups and references values from optical motion capture system and force plate data for walking (top three rows) and running (bottom three rows). The circles show the RMSD for each walking and running cycle of each participant. The bar shows the mean value over all cycles and participants. Joint moments were scaled to bodyweight bodyheight percent (BW BH%) and GRFs were scaled to bodyweight (BW).

Our RMSDs showed similar trends as the coefficient of determination, with setup F generally showing the largest error, while the setups with sensors on the thighs or on the pelvis were most accurate for all variables (Figure 7). The RMSD was highest for setup F for most variables, except for the hip moment for running, where setup FS yielded the highest RMSD. The joint angle RMSD was up to 11 deg higher (running, knee) for setup F than setup FSTP, while the GRF RMSD was up to 0.37 BW higher (running, vertical) and the joint moment RMSD up to 5.5 BW BH% higher (running, ankle). The differences between setup FSTP and the other sparse setups were smaller than those between setup FSTP and setup F. Compared to setup FSTP, setups FP and FSP had the largest increase in joint angle RMSD with 3 deg (hip, walking), while the largest increase was 2.6 deg (walking, knee) for setup FS and 0.8 deg for the setups FT and FTP (running, knee). The joint angle RMSD was even lower than for setup FSTP for setups FS (walking, ankle), FSP (walking and running, ankle), FT (walking, hip and knee, and running, ankle), and FTP (walking, all joints). The GRF RMSD for setup FSTP was up to 0.035 BW lower (running, anterior-posterior) than for setup FP, up to 0.051 BW lower (running, vertical) than for setup FT, up to 0.13 BW lower (running, vertical) than for setup FS, and up to 0.015 BW lower (running, vertical) than setups FSP and FTP. The GRF RMSD was even lower for setups FP (walking, vertical), FT (walking, anterior-posterior), and FTP (running, vertical) than for setup FSTP. The joint moment RMSD was consistently highest in the ankle for running. Compared to setup FSTP, it was up to 2.6 BW BH% higher for setups FP and FT, up to 3 BW BH% higher for setup FS, up to 1.2 BW BH% higher for setup FTP, and up to 0.8 BW BH% higher for setup FSP. The joint moment RMSD for setup FT (walking, hip and knee) was even lower than for setup FSTP. The range of the hip and knee moment RMSD of were similar to those of the ankle moment, while for the coefficient of determination, the difference in range between the hip and knee moment and the ankle moment was larger.

## 4 Discussion

We evaluated optimal control simulations from six different sparse IMU setups by comparing their accuracy against a full lower-body sensor setup and a OMC-based motion analysis. We showed that it is possible to reconstruct sagittal-plane walking and running biomechanics, including spatiotemporal, kinematic and kinetic variables, from sparse inertial sensor setups. We found a good fit between the IMU-based analysis and the OMC-based analysis for most sensor setups with the coefficient of determination showing moderate to strong relationships for all variables and setups except the knee moment for setups F, FP, and FSP, and the hip moment for setups FTP and FSTP, although individual variation was large for most kinetic variables. Furthermore, we found that the sensor setups that included sensors on either the thighs or the pelvis only had small performance drops compared to FSTP, while the differences were larger for setup F for all tested spatiotemporal, kinematic, and kinetic variables and for setup FS in stance time and kinetics, though the results depended on the variable type.

When comparing different sparse IMU setups, we found that in general it is beneficial to place sensors on at least one additional body segment in addition to sensors on the feet, since RMSD improved or remained similar for the spatiotemporal variables, and so did the RMSD and coefficient of determination for most kinetic and kinematic variables (Figures 3, 6, 7). We also found that the optimal sensor setup depends on the application variables of interest and that a full lower-body sensor setup does not always produce the best results. For the spatiotemporal variables, especially for walking, adding sensors to the thighs reduced prediction accuracy (Figure 3), while adding thigh sensors improved similarity of the kinetic and kinematic patterns (Figure 5). Adding a sensor to the pelvis also improved the accuracy of kinematics and kinetics, but not as much as when using thigh sensors. Omitting shank sensors seems reasonable when sensors at the feet and the pelvis or thighs are used since estimation accuracy either only slightly improved or even decreased when adding shank sensors.

We compared different sparse sensor setups to previous work and found higher or similar RMSDs in our work. Previously, we compared the results of the full lower-body setup (Dorschky et al., 2019a), and showed that kinematics and kinetics were comparable to Karatsidis et al. (2019). Weygers et al. (2020) reviewed kinematics estimates from inertial sensors of gait and other movements. We found that our kinematic estimates for all setups are similar to other gait estimates (e.g., Tadano et al., 2013). Similar to our work, RMSD was generally highest for the hip joint angle than and lowest for the ankle joint angle (Tadano et al., 2013; Weygers et al., 2020). Furthermore, the mean RMSD for the knee angle and vertical GRF of setup FP (11 ± 4 deg and 0.3 ± 0.1 BW , respectively) are comparable to those of a similar setup reported by Wouda et al. (2018) ( 5−20 deg for the peak knee extension angle and 0.2−1.3 BW for the peak vertical GRF, who trained several neural networks to output specific kinematic and kinetic variables. We also compared the relative RMSDs of our GRFs estimations to those of Carter et al. (2024), who combined IMUs and pressure sensors to estimate only GRFs. We found slightly higher relative RMSDs, ranging from 3.5% to 23% for the anterior-posterior GRF, compared to 0.8%–8.8%, and from 2.0% to 25% for the vertical direction, compared to 1.3%–17.3%. These comparisons highlight that our approach using optimal control is as accurate as other commonly used approaches, while it allows for different biomechanical variables to be estimated simultaneously. However, inference of machine learning models is generally fast, while our simulations require half an hour to 2 h to solve.

The estimated joint moments of the reconstruction were surprising, specifically the large range in coefficient of determination for the hip and knee moment. This large range can be explained by the differences between the processing of the OMC and IMU data. The processing of the OMC data included filtering, followed by inverse kinematics and inverse dynamics (Winter, 2009) with a different underlying model. It might be that the low-pass cut-off frequency in our filter was too high, allowing for more high frequency signal to pass than feasible in the optimal control simulation. This would especially affect the coefficient of determination but not the RMSD, as we observed in our results. Furthermore, the general differences in processing yield that the kinetics and kinematics of the OMC result cannot be reproduced exactly in an optimal control simulation. This comparison is therefore challenging, since the exact ground truth is unknown, as joint moments and joint angles cannot be measured directly. To remove most differences between the IMU- and OMC-based analysis, and make the comparison as direct as possible, the optimal control simulations tracking IMU data could be compared against optimal control simulations tracking marker and GRF (Nitschke et al., 2023). Furthermore, the large range in the coefficient of determination could also indicate a difference in accuracy between individual participants. For example, personalization of the musculoskeletal model might improve the results, since the general musculoskeletal model that was used might represent some individuals better than others. Therefore, it is important to further analyse differences between individuals as well to gain insights into the causes of the large range in the coefficient of determination.

Furthermore, the setup of the optimal control problem used for the biomechanical gait reconstructions could also affect the accuracy of the simulations. For example, contact between the ground and our musculoskeletal model is created by a rigid foot with two penetration-based contact points, one at the heel and one at the toe, which is a simplification of foot-ground contact as it happens in practice. These model simplifications cause different kinetics to be associated with certain kinematics for the musculoskeletal model than for the experimental participants, such that the kinetics as recorded by and estimated from OMC cannot be achieved. The effect of this foot-ground model on the ankle moment accuracy was already observed for the full lower-body sensor setup (Dorschky et al., 2019b). It should be investigated if kinetic estimations, especially horizontal GRF estimations, can be improved with a more sophisticated foot-ground contact model.

Another choice in the optimal control setup is the weighting between the effort and tracking objective. We chose our weighting based on Dorschky et al. (2019b) and normalized it to the number of sensors to ensure that the objective value was similar for all setups. This weight term could still be tuned individually for each sensor setup, and could even be tuned for each sensor. For example, a larger weight on the pelvis or thigh sensors could improve accuracy, especially in a sparse sensor setup, because we found that its inclusion in the sensor setup is important. Additional objective terms could be considered in future work to compensate for missing information in the IMU data. For example, Falisse et al. (2019) found that a multi-objective cost function minimizing metabolic energy rate, muscle activations, and joint accelerations predicted human-like walking patterns without tracking measured data.

There are also different approaches to track data in the optimal control problem. Here, we chose to track the mean and variance over 10 gait cycles for each participant. Instead, we could have also created simulations for each gait cycle individually (Nitschke et al., 2024). This decision depends on the motivation for creating the simulations. When the goal is to analyze a longitudinal parameter, such as repeated loading or fatigue, the benefit of tracking an average gait cycle is that the simulation does not reflect natural variation. From a technical perspective, averaging and then simulating is also faster than first simulating and then averaging. An additional advantage is that we adjust the weighting to the natural variance of the movement, since a large variance reduces the objective weighting and a small variance increases it. Due to this, parts of the movement with little variance are tracked more closely that parts with much variance. However, when the goal is to provide biofeedback, for example during training, it makes more sense to provide this for each gait cycle individually and therefore simulate individual gait cycles.

We have shown that it is possible to create sagittal-plane reconstructions of walking and running, and estimate kinetics and kinematics from sparse sensor setups. We were able to create reconstructions with only three sensors (on the feet and the pelvis) or only four sensors (on the feet and the thighs) that had only a few noticeable differences to those created with a full lower-body sensor setup. These results imply that the improved usability of having only three or four sensors does not lead to a considerable performance drop of the movement reconstructions. Our results support previous work that showed that, while such movement reconstructions are possible using only foot sensors, results are worse in this approach (Dorschky et al., 2019b). In contrast, deep neural networks that directly map foot-worn sensor data to lower-leg kinematics have shown promising results (Hossain et al., 2022). They reported mean RMSDs for sagittal-plane joint angles of 4 to 5 deg , whereas our tracking simulations using only foot-worn IMUs resulted in mean RMSDs of 4 to 13 deg . One interesting direction for future work would be to combine deep learning and physics-based optimization to obtain physically correct human motion from deep neural networks (Yi et al., 2022; Dorschky et al., 2020).

Different sensor setups and processing approaches could be investigated to further optimize both usability and accuracy. For example, a sensor setup with three sensors on the shanks and the pelvis could be considered. We chose the setup with sensors on the feet to include as many body segments as possible between the sensors, because no information would be available about the feet when sensors are placed on the shank. Furthermore, it might be possible to increase sparsity by placing sensors asymmetrically on different body segments when symmetric motions are recorded, to avoid double recording of the same signal, e.g., on the left shank and right thigh. Such setups could be further investigated using an observability analysis to investigate the exact working conditions in which kinematic and kinetic variables can be inferred from simulated inertial measurement data. So far, such an analysis has been performed using recurrent neural networks (RNNs) to estimate the observability of kinematics on simplified mechanical linkages (Bachhuber et al., 2022), as well as for sparse inertial motion tracking (Bachhuber et al., 2023). Nevertheless, in future work such an RNN-based observability analysis might be used to study the observability of both kinematic and kinetic variables in sparse IMU setups for realistic biomechanical kinematic chains, such as we used here. To further improve usability, our method’s robustness against uncertainty in the sensor placement and orientation estimation should be investigated. In our experiment, the sensor positions were measured by hand, which might not always be feasible in practice. These positions could also be estimated as part of an optimal control problem or neural network output. The orientation estimation currently requires calibration movements to be performed to identify the direction of a vector normal to the sagittal plane. While these movements would not be necessary in an ideal case, they are easy to perform by the user. It could be investigated if the orientation can be estimated from walking and running directly, as both are also mainly sagittal plane movements.

These results will help those interested in studying motion “in the wild”, or outside of the lab environment, since we have shown that using sparse sensor setups will not necessarily reduce accuracy of the resulting simulations. Sparse sensor setups with high accuracy enable capturing of real-life data with reduced burden on patients compared to a full sensor setup. This real-life data can then support decision-making by providing objective data complementing subjective clinical scores. In particular, sensor-based recordings of movement patterns in different patient cohorts are beneficial to deeply understand intra- and interday variability of motor impairments of chronic diseases. This understanding is of major importance to characterize subgroups of patients or develop personalized approaches in order to evaluate disease progression and therapy response. The real-life data could also help detect changes early in the disease course, which is highly relevant for applying disease-stage-specific therapies. Future studies should evaluate the accuracy, usability as well as the technical and clinical validity of a sparse sensor setup in patients with movement disorders. This would set the basis for robust and valid sensor-based data recordings in real-life with the long-term goal to improve daily care and quality of life of patients using digital technologies.

## 5 Conclusion

In conclusion, our work shows that we can accurately perform a comprehensive sagittal-plane motion analysis with sparse sensor setups. We conclude that a comprehensive analysis including spatiotemporal, kinematic, and kinetic variables can best be performed with a sparse sensor setup that include sensors on the feet, the pelvis, and the thighs. We found that different setups performed better for different types of variables. A setup with feet and pelvis sensors was as accurate as the full lower-body setup for spatiotemporal and kinetic variables, while a setup with feet and thigh sensors was as accurate as the full lower-body setup for kinematic and kinetic variables, with a performance drop for the ankle moment up to 2 BW BH% using both sensor setups during running. Therefore, when a comprehensive analysis is not necessary, the sparse sensor setup can be optimized for each application.

This study serves as a first step towards validating sparse inertial sensor sets for monitoring movements “in the wild.” Future validation studies should ensure that the processing between the IMU-based motion analysis and the ground truth motion analysis is as similar as possible, to avoid including processing differences in the results. This way, validity and usability of sparse sensor setups can be further evaluated for specific applications in order to allow the use of gait analysis in a wide spectrum of clinical and sports applications.

## Data Availability

The dataset presented in this study can be found in an online repository. The repository and accession number can be found below: https://doi.org/10.5281/zenodo.11522050.

## Footnotes

1 We use the notation [minimum value;maximum value] to show the range of the root mean square deviation over all simulations of all participants.
