# Supplementary material for: Comparing sparse inertial sensor setups for sagittal-plane walking and running reconstructions
Source: Front Bioeng Biotechnol. 2025 Feb 19;13:1507162. doi: 10.3389/fbioe.2025.1507162 (PMC11879983; doi:10.3389/fbioe.2025.1507162)

# Report of P07 fastwalking setup FSP

June 10, 2024

## 1 Solver

### 1.1 Solver Status

- Status ID: Solve_Succeeded
- Status Message: Optimal Solution Found
- Number of iterations: 1308
- CPU time: 00:36:23 (HH:MM:SS)

### 1.2 Solver Settings

- Solver: IPOPT
- tol: 0.0001
- max_iter: 20000
- constr_viol_tol: 0.001
- compl_inf_tol: 0.001
- acceptable_tol: 1e-06
- bound_frac: 0.001
- bound_push: 0.001
- hessian_approximation: limited-memory
- check_derivatives_for_naninf: no
- mu_strategy: adaptive
- linear_solver: mumps
- print_level: 5
- print_timing_statistics: yes
- For all other options, default values were used.

# 2 Problem

## 2.1 General Information

- Model: Gait2dc
- Number of nodes: 100
- Symmetry: false
- Euler Method: BE
- Translation speed: 1.642 (m/s)
- Movement duration: 0.998 (s)
- Metabolic cost: 3.059 (J/m/kg)
- Objective Terms:

| name | weightedValue | weight | unweightedValue |
|---|---|---|---|
| regTerm | 2.594041e-03 | 1.000000e-05 | 2.594041e+02 |
| effortTermMuscles | 3.666291e+00 | 3.000000e+02 | 1.222097e-02 |
| trackAcc | 3.744242e+00 | 2.000000e+00 | 1.872121e+00 |
| trackGyro | 2.928210e+00 | 1.000000e+00 | 2.928210e+00 |

  tracked Variables:

  - Acc: foot_l, foot_r, pelvis, tibia_l, tibia_r
  - Gyro: foot (left and right), pelvis, tibia (left and right)

- Constraint Terms:

| name | normc |
|---|---|
| dynamicConstraints | 7.604143e-08 |
| periodicityConstraint | 4.965068e-16 |

## 2.2 GRF

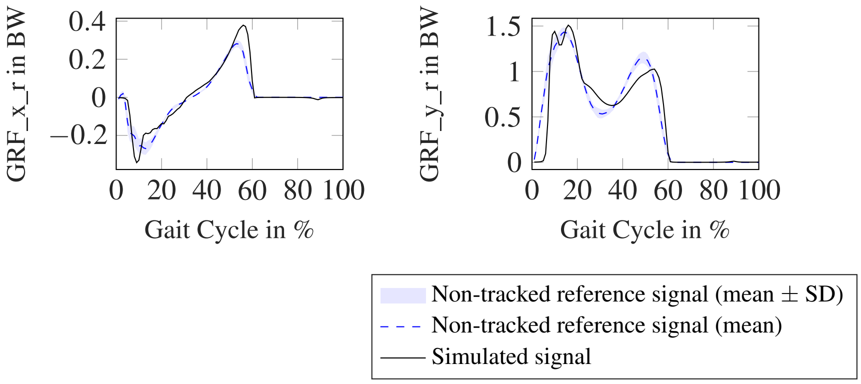

## 2.3 acc

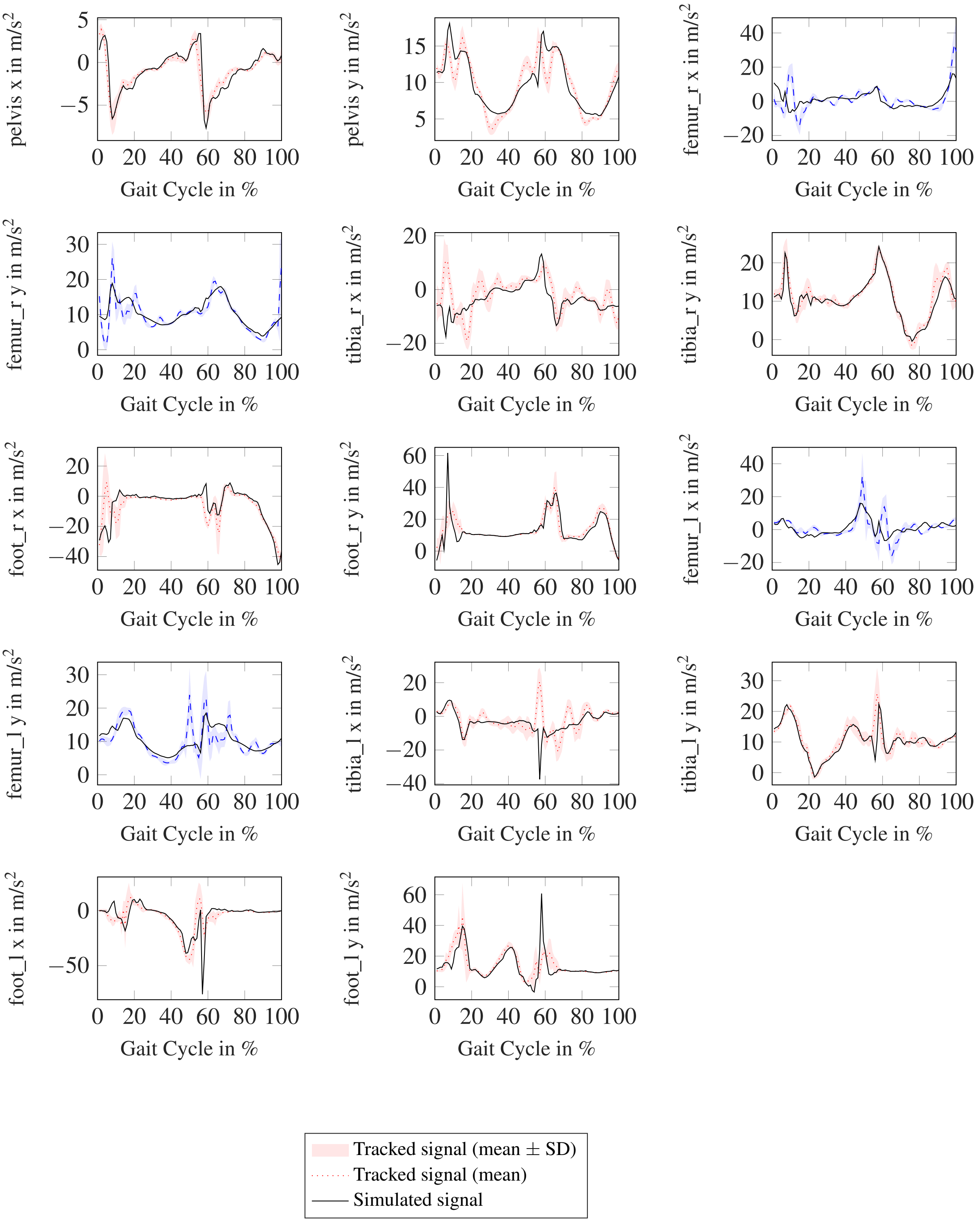
pelvis x in m/s$^2$
pelvis y in m/s$^2$
femur_r x in m/s$^2$
femur_r y in m/s$^2$
tibia_r x in m/s$^2$
tibia_r y in m/s$^2$
foot_r x in m/s$^2$
foot_r y in m/s$^2$
femur_l x in m/s$^2$
femur_l y in m/s$^2$
tibia_l x in m/s$^2$
tibia_l y in m/s$^2$
foot_l x in m/s$^2$
foot_l y in m/s$^2$
Gait Cycle in %
Tracked signal (mean ± SD)
Tracked signal (mean)
Simulated signal

## 2.4 angle

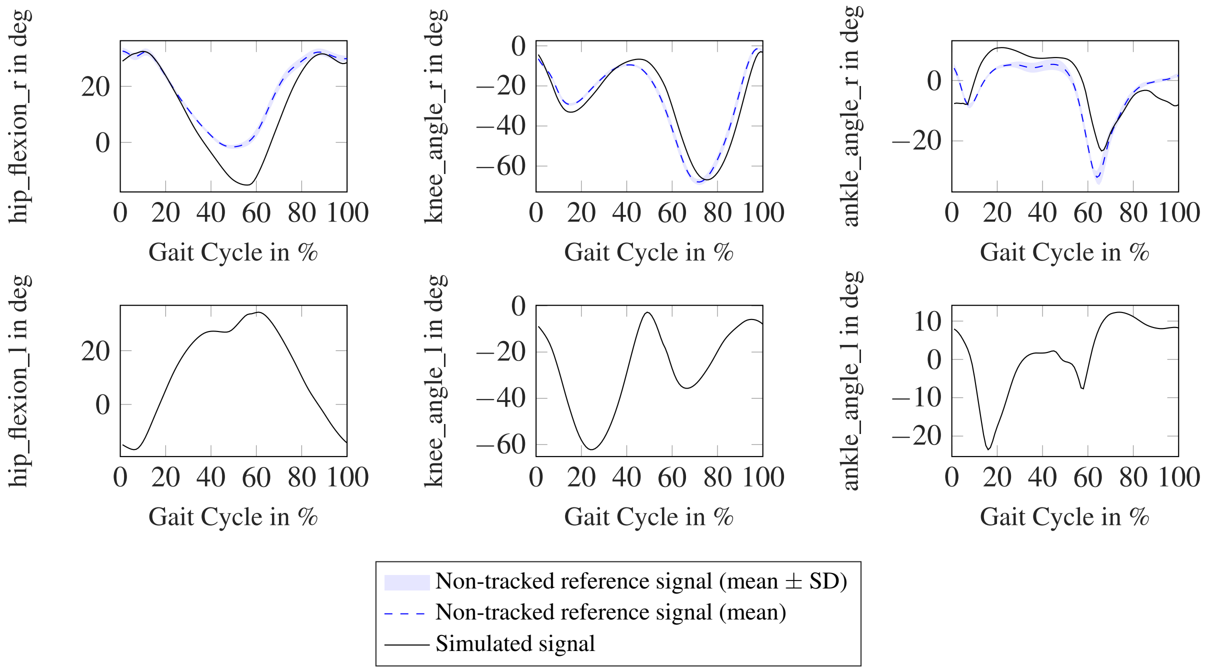


## 2.5 gyro

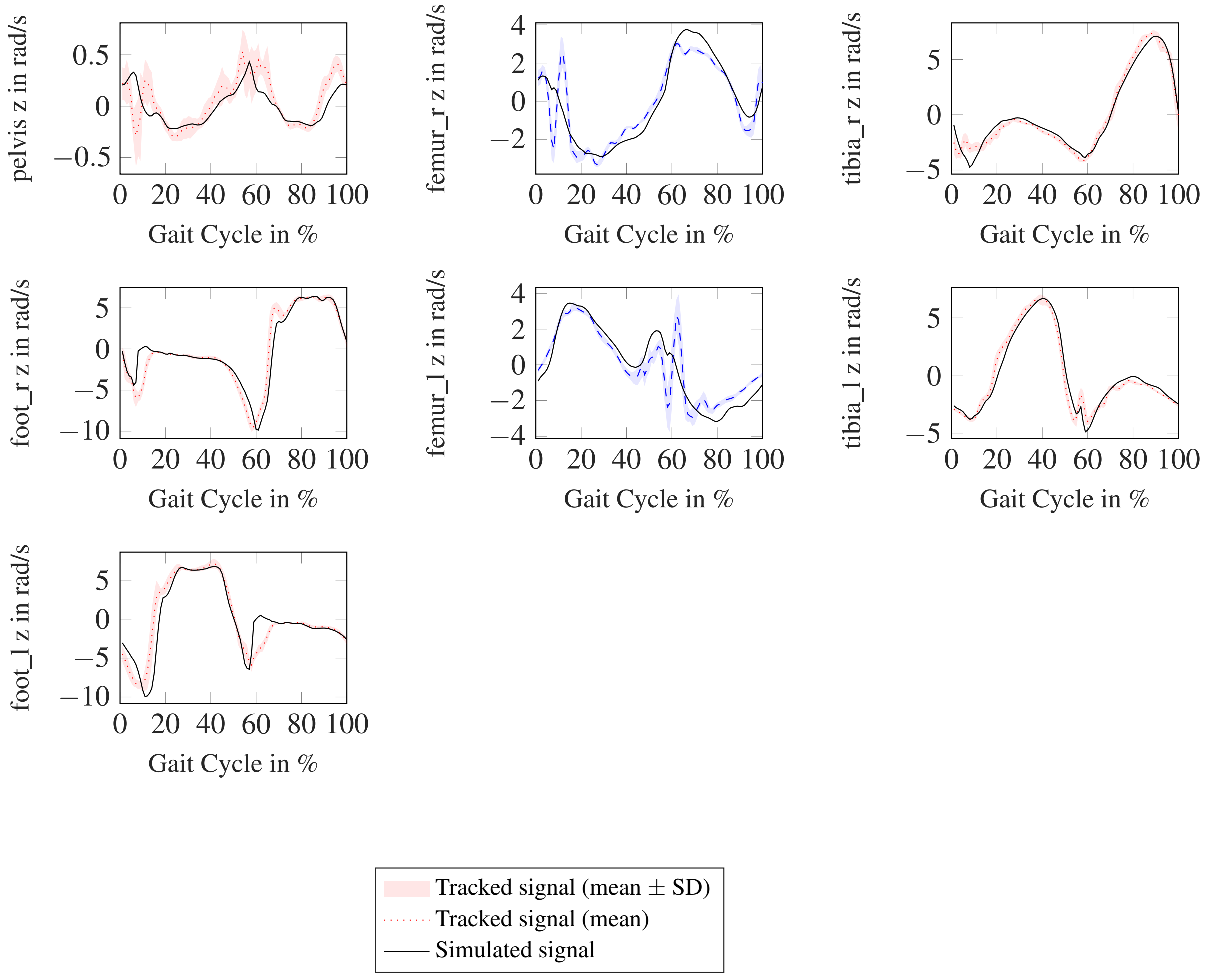

## 2.6 moment

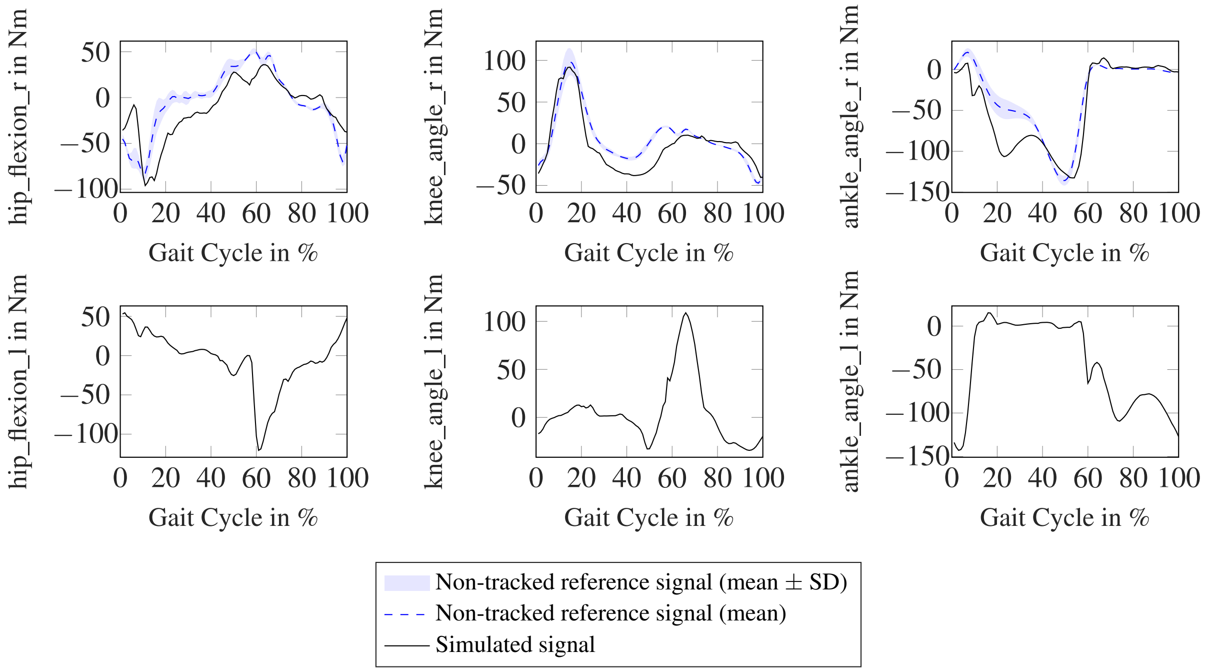


## 2.7 muscleForce

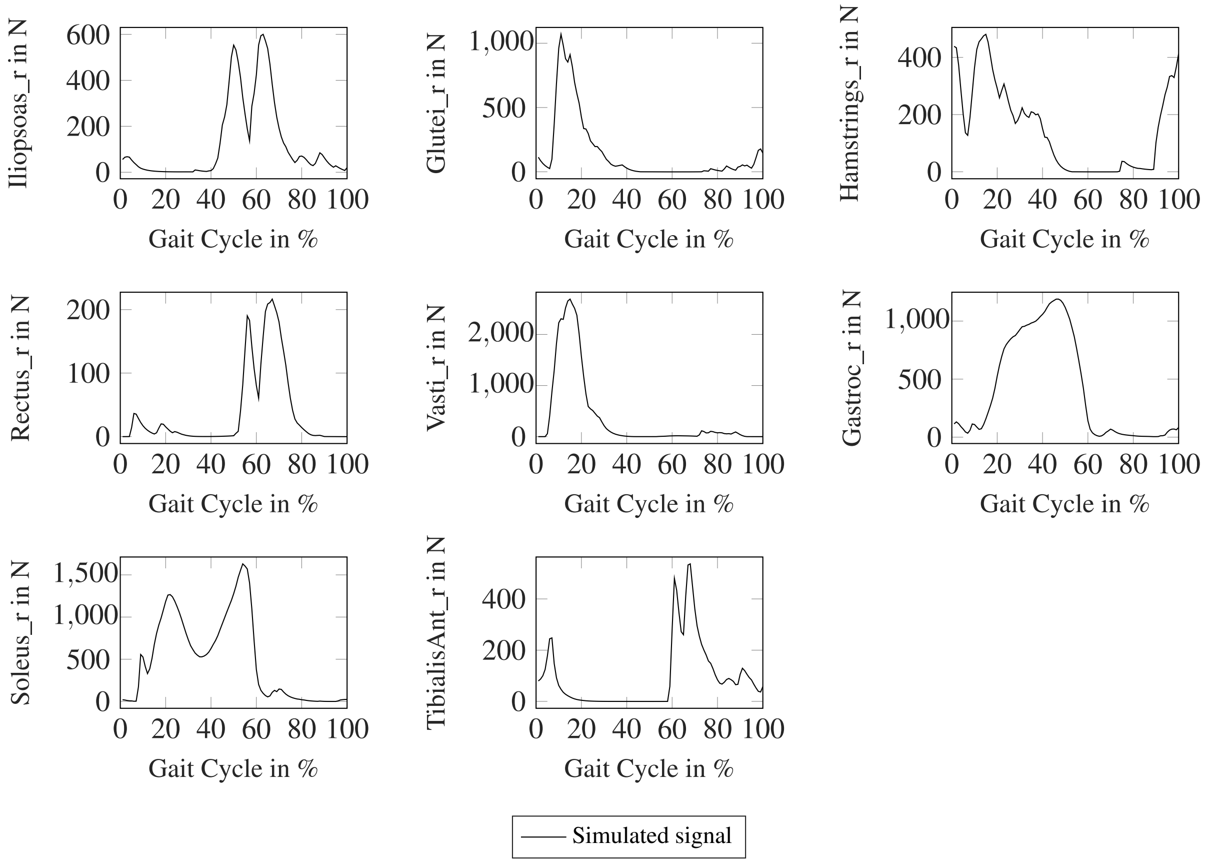

Supplement: Supplementary file 3 [file DataSheet8.zip › P07_fastwalking_setup_FSP_report.pdf]
