# Supplementary material for: Comparing sparse inertial sensor setups for sagittal-plane walking and running reconstructions
Source: Front Bioeng Biotechnol. 2025 Feb 19;13:1507162. doi: 10.3389/fbioe.2025.1507162 (PMC11879983; doi:10.3389/fbioe.2025.1507162)

# Report of P07 slowrunning setup FT

June 10, 2024

## 1 Solver

### 1.1 Solver Status

- Status ID: Solve_Succeeded
- Status Message: Optimal Solution Found
- Number of iterations: 1493
- CPU time: 00:35:57 (HH:MM:SS)

### 1.2 Solver Settings

- Solver: IPOPT
- tol: 0.0001
- max_iter: 20000
- constr_viol_tol: 0.001
- compl_inf_tol: 0.001
- acceptable_tol: 1e-06
- bound_frac: 0.001
- bound_push: 0.001
- hessian_approximation: limited-memory
- check_derivatives_for_naninf: no
- mu_strategy: adaptive
- linear_solver: mumps
- print_level: 5
- print_timing_statistics: yes
- For all other options, default values were used.

# 2 Problem

## 2.1 General Information

- Model: Gait2dc
- Number of nodes: 100
- Symmetry: false
- Euler Method: BE
- Translation speed: 3.260 (m/s)
- Movement duration: 0.712 (s)
- Metabolic cost: 4.190 (J/m/kg)
- Objective Terms:

| name | weightedValue | weight | unweightedValue |
|---|---|---|---|
| regTerm | 5.983998e-03 | 1.000000e-05 | 5.983998e+02 |
| effortTermMuscles | 4.096472e+00 | 3.000000e+02 | 1.365491e-02 |
| trackAcc | 2.846041e+00 | 2.000000e+00 | 1.423021e+00 |
| trackGyro | 1.243813e+00 | 1.000000e+00 | 1.243813e+00 |

tracked Variables:

- Acc: femur_l, femur_r, foot_l, foot_r
- Gyro: femur (left and right), foot (left and right)

- Constraint Terms:

| name | normc |
|---|---|
| dynamicConstraints | 1.152533e-07 |
| periodicityConstraint | 4.440892e-16 |

## 2.2 GRF

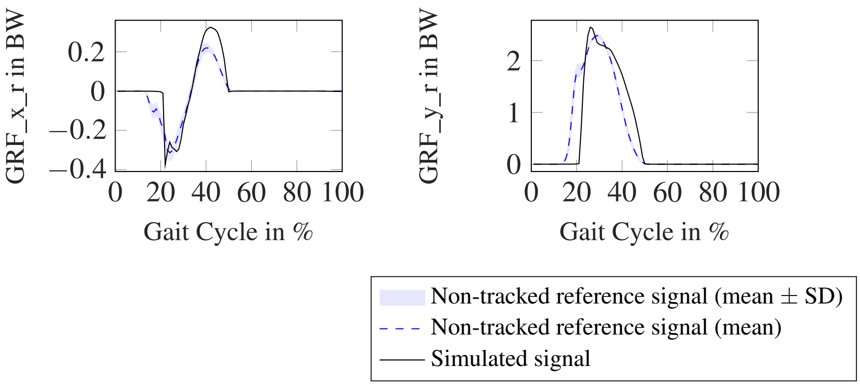

## 2.3 acc

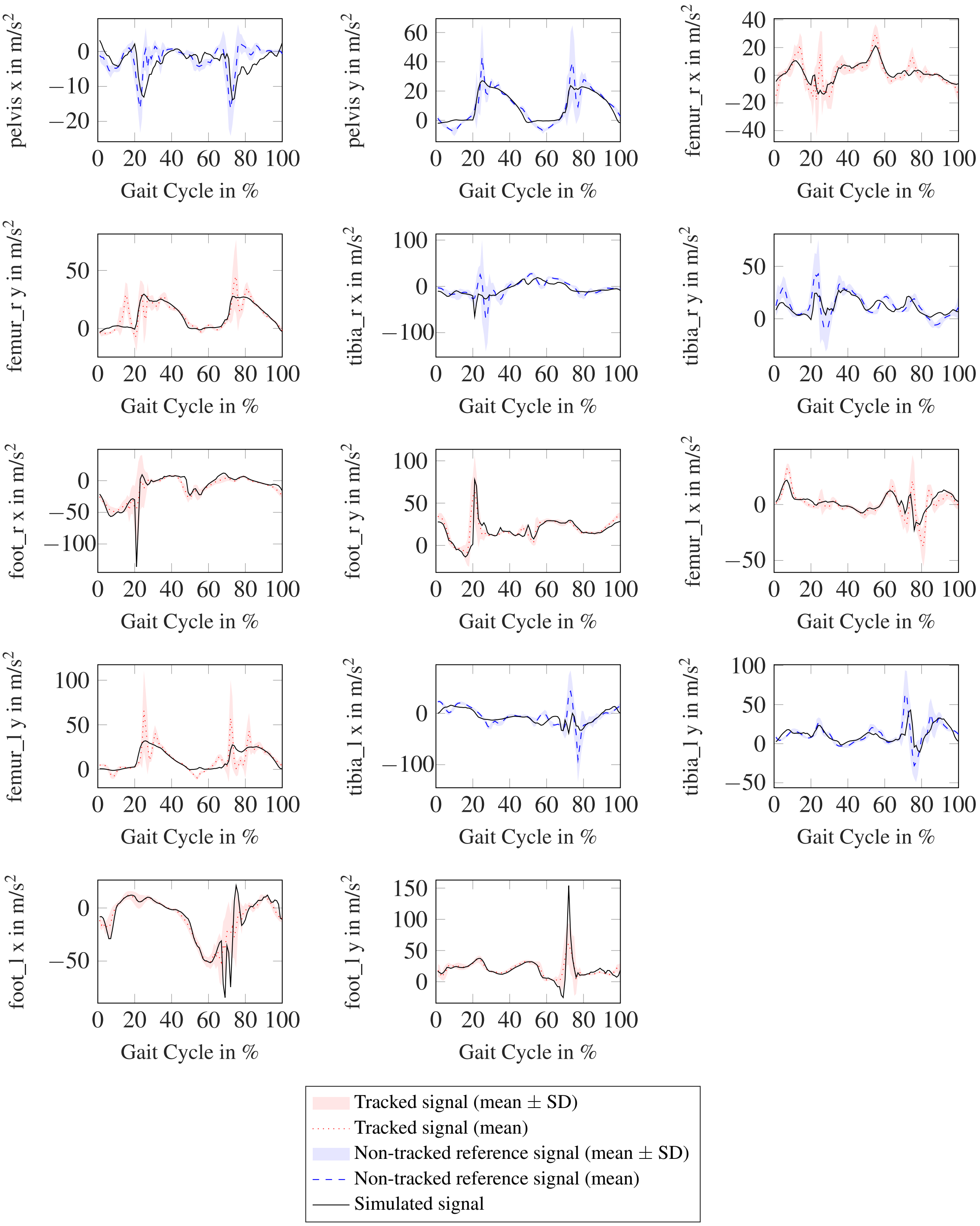
pelvis x in m/s$^2$
pelvis y in m/s$^2$
femur_r x in m/s$^2$
femur_r y in m/s$^2$
tibia_r x in m/s$^2$
tibia_r y in m/s$^2$
foot_r x in m/s$^2$
foot_r y in m/s$^2$
femur_l x in m/s$^2$
femur_l y in m/s$^2$
tibia_l x in m/s$^2$
tibia_l y in m/s$^2$
foot_l x in m/s$^2$
foot_l y in m/s$^2$
Gait Cycle in %
Tracked signal (mean ± SD)
Tracked signal (mean)
Non-tracked reference signal (mean ± SD)
Non-tracked reference signal (mean)
Simulated signal

## 2.4 angle

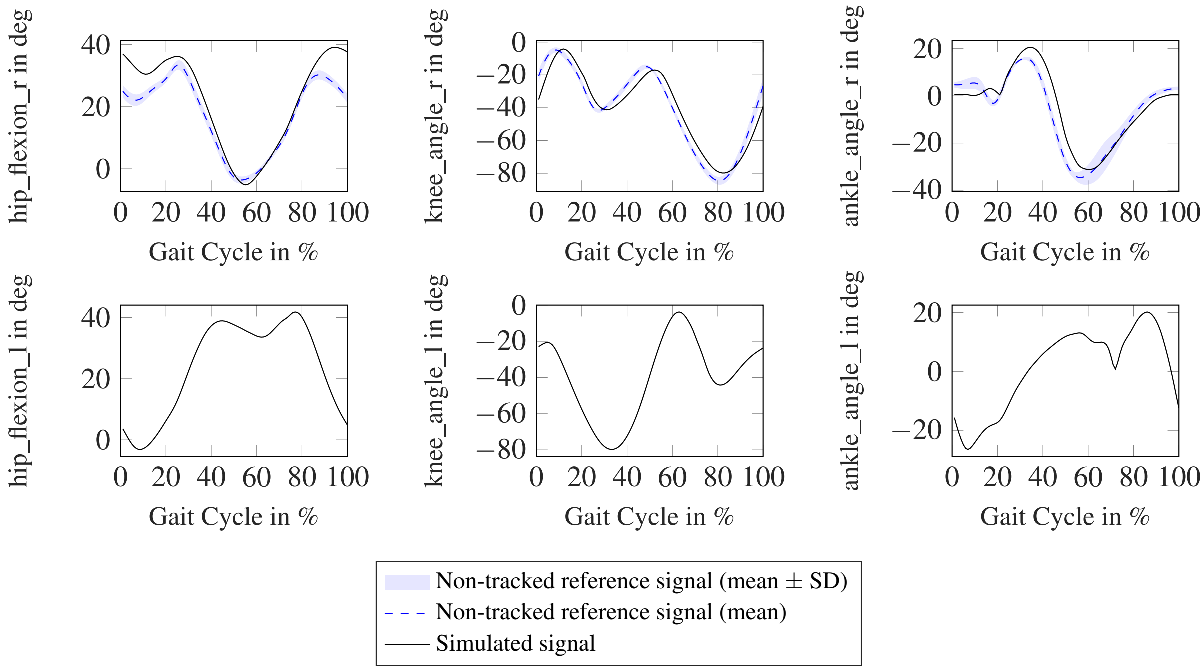


## 2.5 gyro

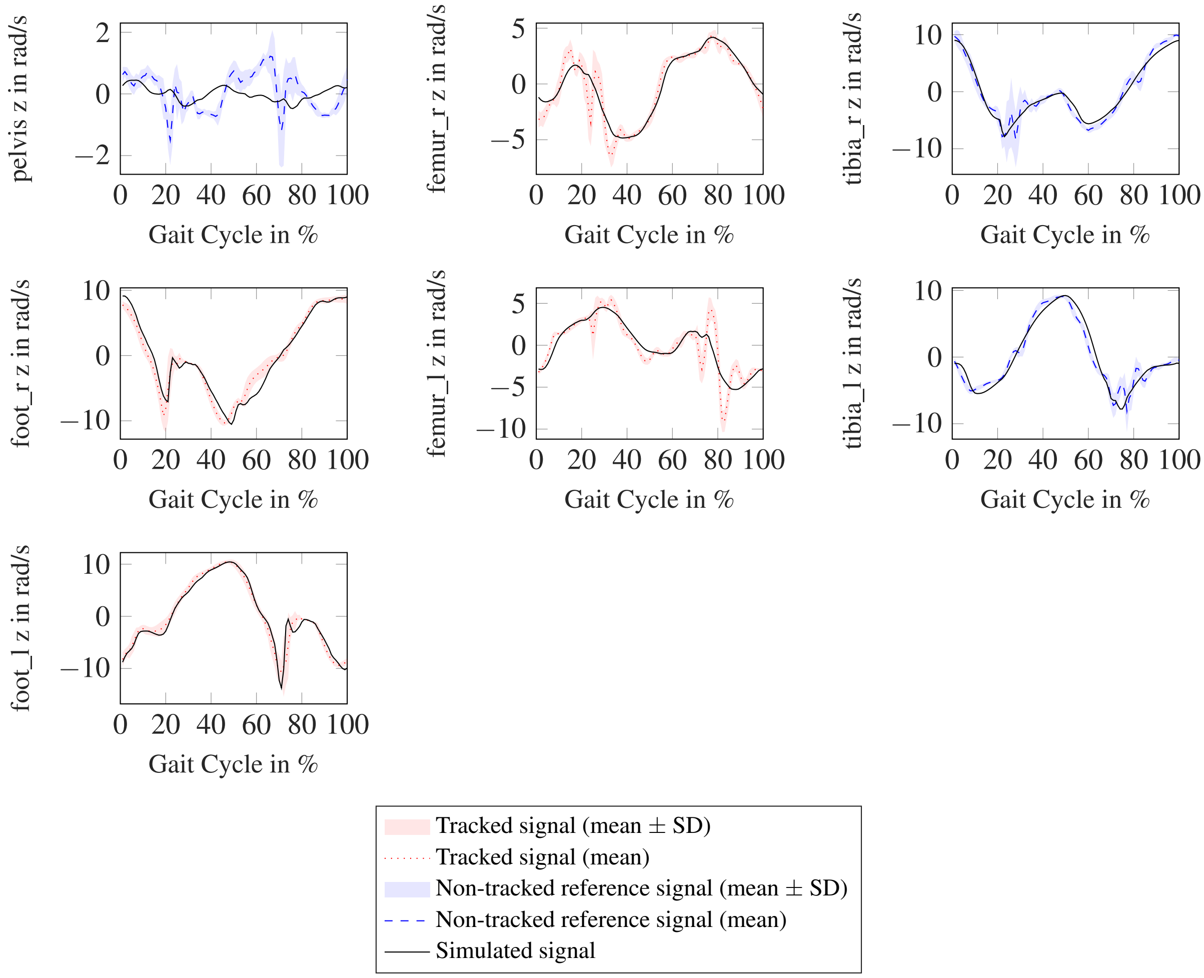

## 2.6 moment

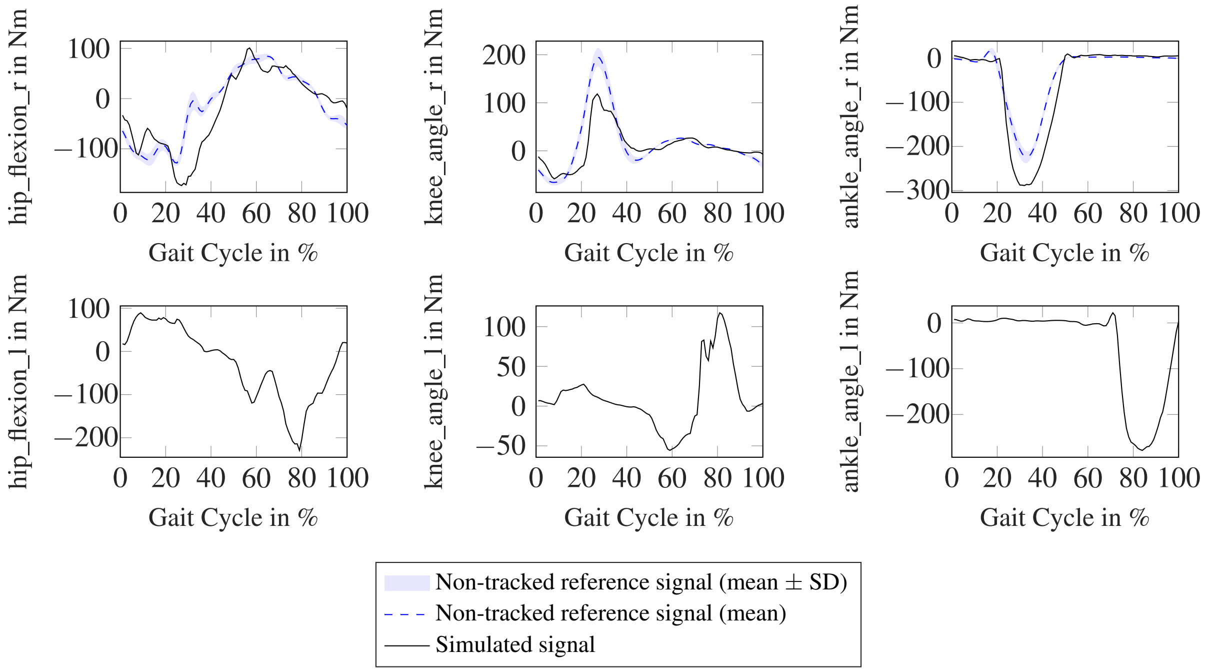


## 2.7 muscleForce

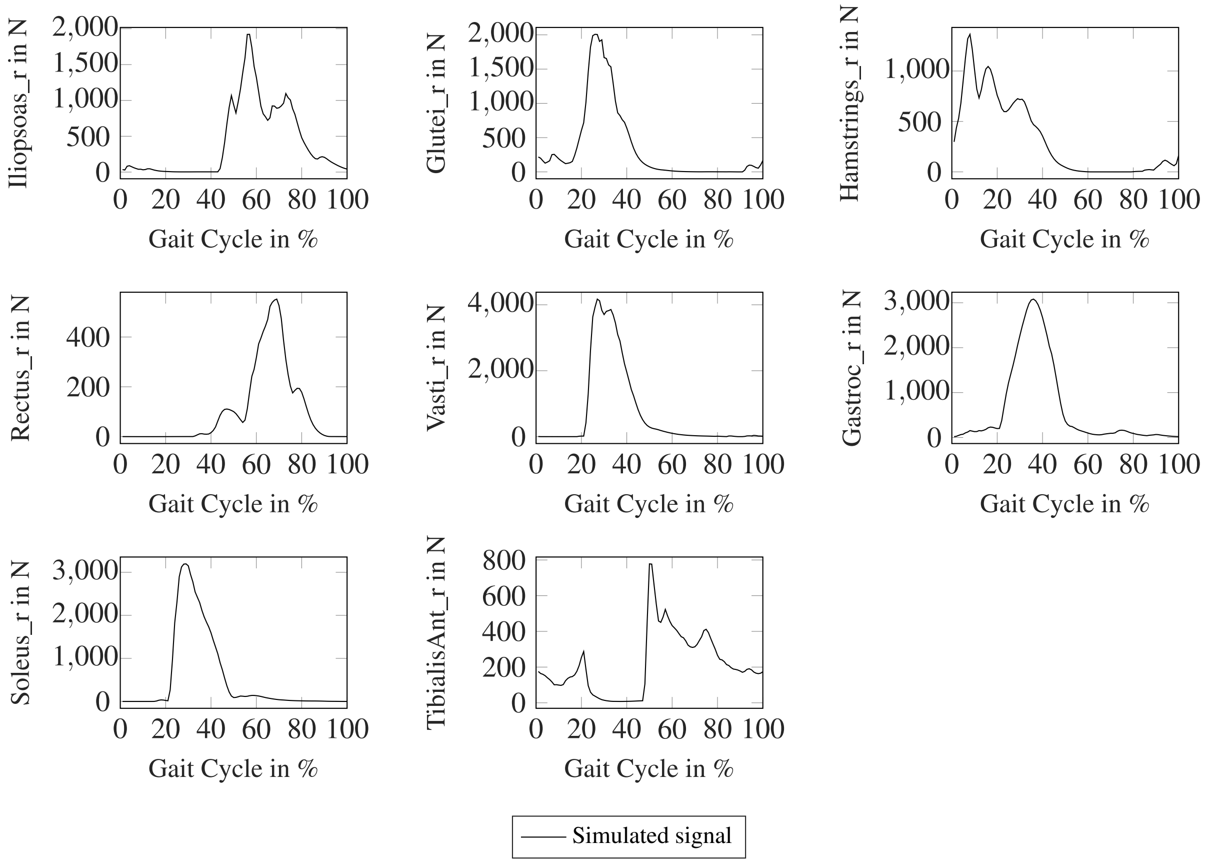

Supplement: Supplementary file 3 [file DataSheet8.zip › P07_slowrunning_setup_FT_report.pdf]
