# Supplementary material for: Comparing sparse inertial sensor setups for sagittal-plane walking and running reconstructions
Source: Front Bioeng Biotechnol. 2025 Feb 19;13:1507162. doi: 10.3389/fbioe.2025.1507162 (PMC11879983; doi:10.3389/fbioe.2025.1507162)
Supplement: Supplementary file 8 [file DataSheet1.pdf]

Supplementary Material: Averaged Sagittal Plane Inertial Sensor Signals and Individual Reports

Figure S1 and Figure S2 show the inertial sensor signals of the simulations and the reference signals for walking and running, respectively. The signals were averaged over all participants and speeds for each of the tested sensor setups. The sensor setups are abbreviated using the first letter of the segments equipped with a sensor: F-feet, S-shanks, T-thighs, P-pelvis. The setup FSTP corresponds to a full lower-body sensor setup with seven inertial measurement units.

The additional PDFs report the results of the individual trials, for each participant, condition, and sensor setup, according to the name of the the file. Each PDF contains the following:

1. Solver information
2. Problem information
   a. General problem settings and values related to the optimal result.
   b. Ground reaction forces (simulation and reference signal)
   c. Accelerations (simulation, reference, and tracked signal)
   d. Joint angles (simulation and reference signal)
   e. Gyroscope (simulation, reference, and tracked signal)
   f. Joint moment (simulation and reference signal)
   g. Muscle Force (simulation signal)

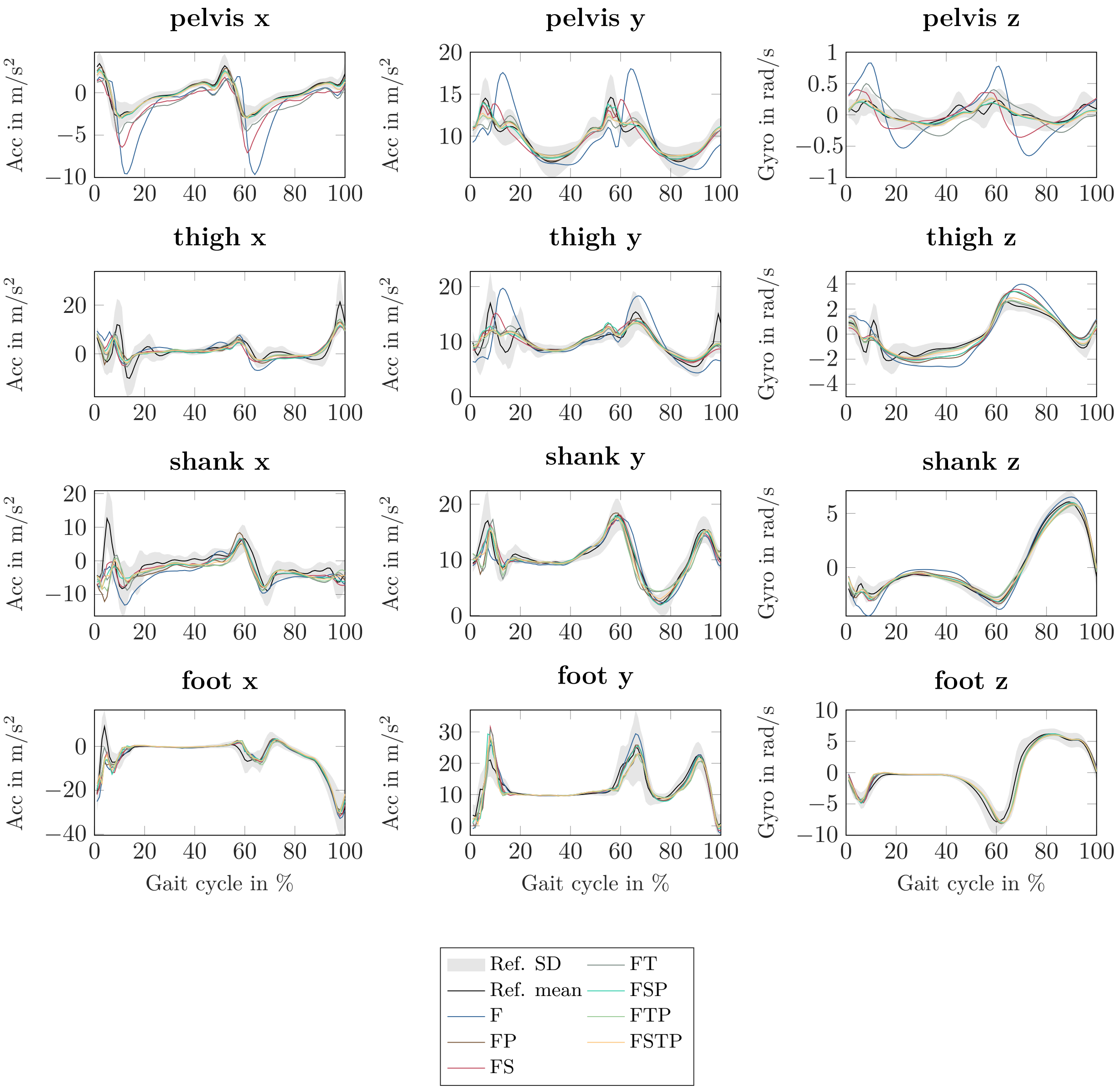

Figure S1. Sagittal-plane inertial sensor signals of the right lower limb for walking at all speeds, from the different inertial measurement unit setups (colored lines) and the references values from optical motion capture system and force plate data (mean: black line, standard deviation: grey fill). All lines represent the mean over all participants from right heel strike to right heel strike.

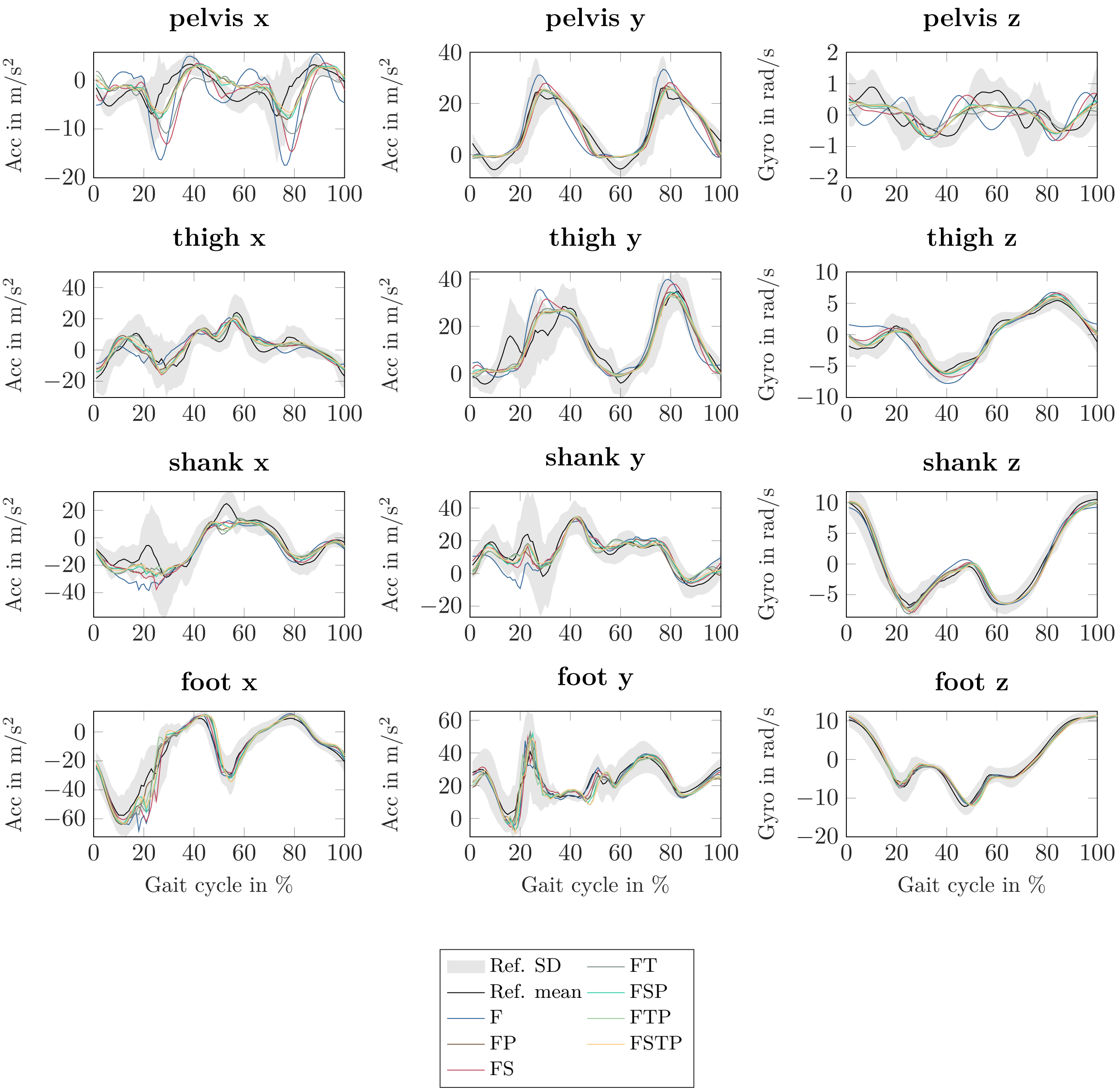

Figure S2. Sagittal-plane inertial sensor signals of the right lower limb for running at all speeds, from the different inertial measurement unit setups (colored lines) and the references values from optical motion capture system and force plate data (mean: black line, standard deviation: grey fill). All lines represent the mean over all participants from left toe off to left toe off.
