# Supplementary material for: Comparing sparse inertial sensor setups for sagittal-plane walking and running reconstructions
Source: Front Bioeng Biotechnol. 2025 Feb 19;13:1507162. doi: 10.3389/fbioe.2025.1507162 (PMC11879983; doi:10.3389/fbioe.2025.1507162)

Report of P01 slowrunning setup FTP

June 9, 2024

1 Solver

1.1 Solver Status

- Status ID: Solve_Succeeded
- Status Message: Optimal Solution Found
- Number of iterations: 581
- CPU time: 00:17:02 (HH:MM:SS)

1.2 Solver Settings

- Solver: IPOPT
- tol: 0.0001
- max_iter: 20000
- constr_viol_tol: 0.001
- compl_inf_tol: 0.001
- acceptable_tol: 1e-06
- bound_frac: 0.001
- bound_push: 0.001
- hessian_approximation: limited-memory
- check_derivatives_for_naninf: no
- mu_strategy: adaptive
- linear_solver: mumps
- print_level: 5
- print_timing_statistics: yes
- For all other options, default values were used.

2 Problem

2.1 General Information

- Model: Gait2dc
- Number of nodes: 100
- Symmetry: false
- Euler Method: BE
- Translation speed: 2.834 (m/s)
- Movement duration: 0.771 (s)
- Metabolic cost: 4.085 (J/m/kg)
- Objective Terms:

name	weightedValue	weight	unweightedValue
regTerm	5.749714e-03	1.000000e-05	5.749714e+02
effortTermMuscles	4.277358e+00	3.000000e+02	1.425786e-02
trackAcc	4.678976e+00	2.000000e+00	2.339488e+00
trackGyro	1.843275e+00	1.000000e+00	1.843275e+00

tracked Variables:

- Acc: femur_l, femur_r, foot_l, foot_r, pelvis
- Gyro: femur (left and right), foot (left and right), pelvis

- Constraint Terms:

name	normc
dynamicConstraints	8.745603e-08
periodicityConstraint	7.691851e-16

2.2 GRF

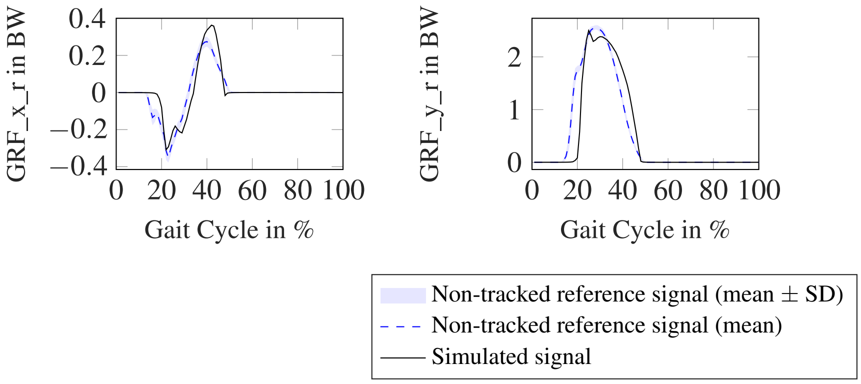

2.3 acc

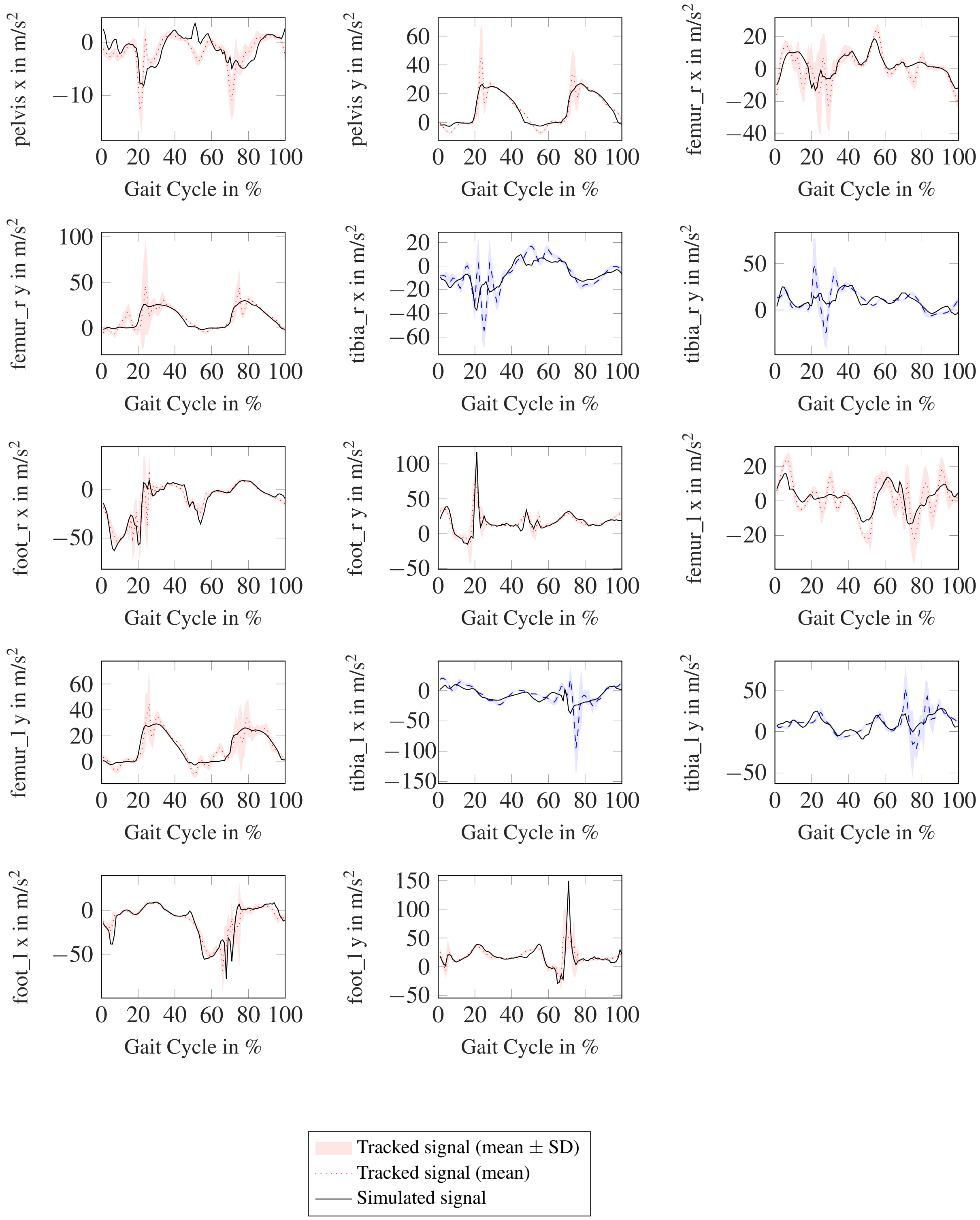
pelvis x in m/s²
pelvis y in m/s²
femur_r x in m/s²
femur_r y in m/s²
tibia_r x in m/s²
tibia_r y in m/s²
foot_r x in m/s²
foot_r y in m/s²
femur_l x in m/s²
femur_l y in m/s²
tibia_l x in m/s²
tibia_l y in m/s²
foot_l x in m/s²
foot_l y in m/s²
Gait Cycle in %
Tracked signal (mean ± SD)
Tracked signal (mean)
Simulated signal

2.4 angle

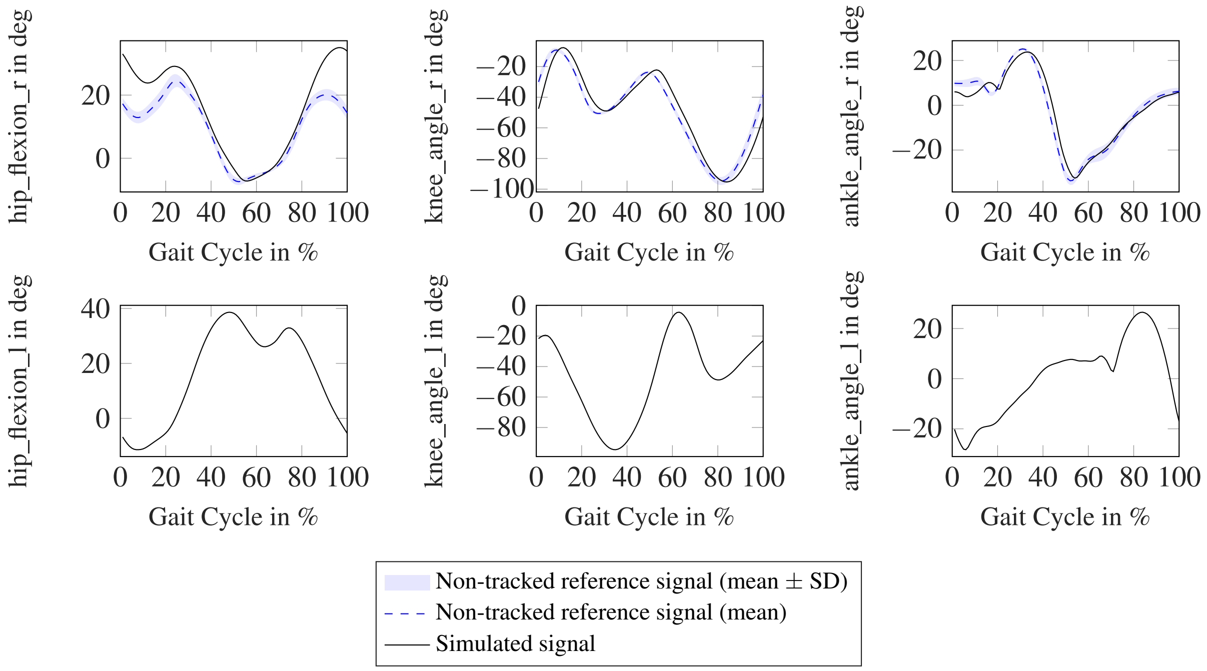

2.5 gyro

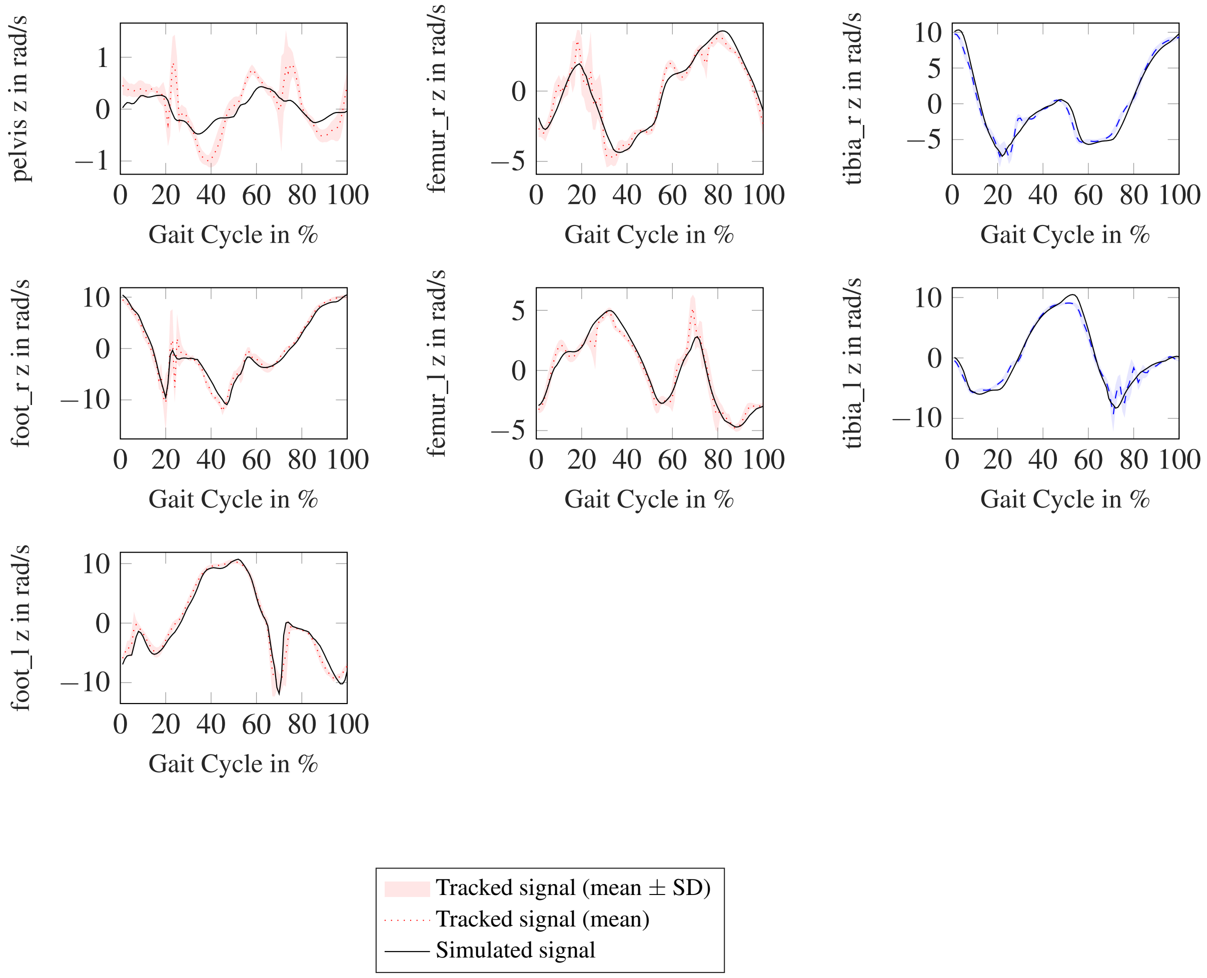

2.6 moment

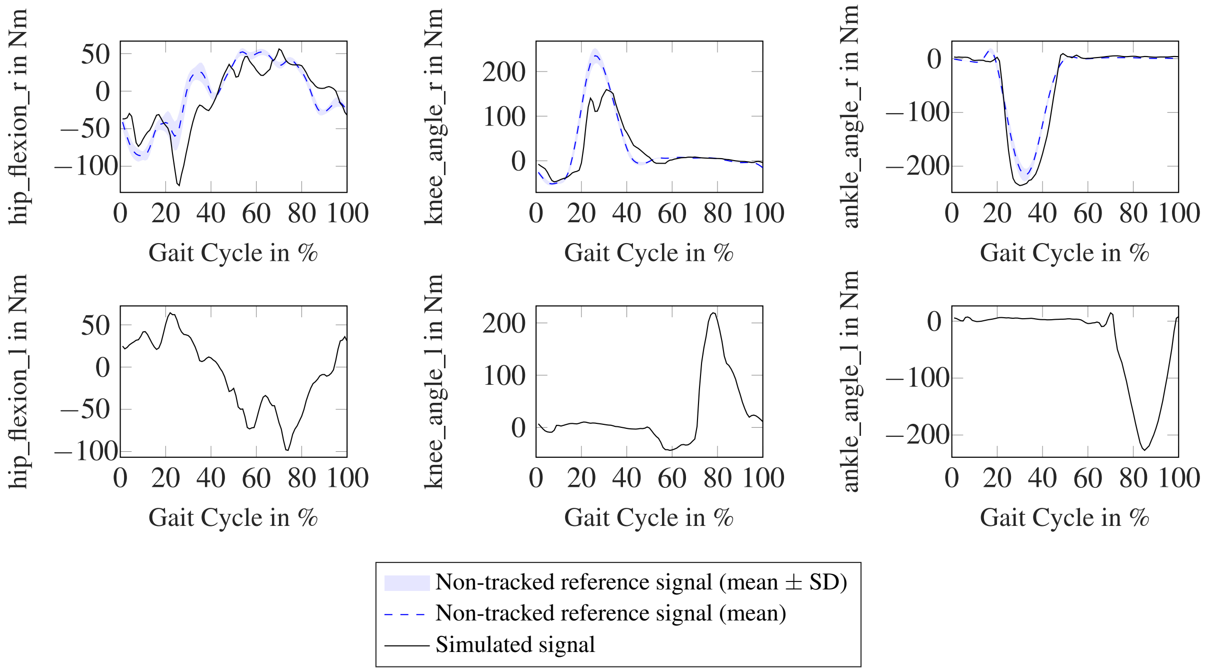

2.7 muscleForce

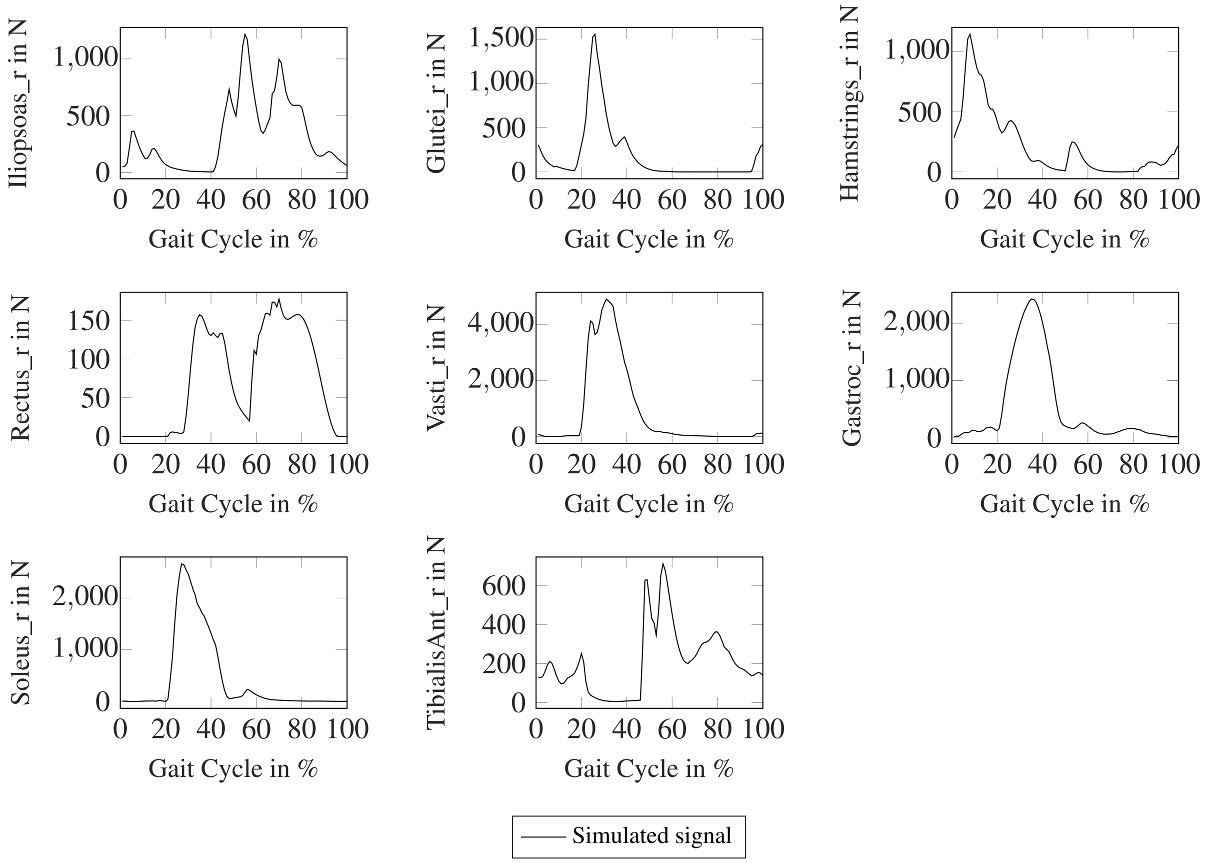

Supplement: Supplementary file 9 [file DataSheet2.zip › P01_slowrunning_setup_FTP_report.pdf]
